# Genome-Wide Characterization of the TGF-β Gene Family in Donkey (*Equus asinus*) Reveals Lineage-Specific Gene Duplications and Deleterious Mutations

**DOI:** 10.3390/ani16132028

**Published:** 2026-07-02

**Authors:** Tanveer Nasir, Muhammad Tariq, Mohamed Tharwat, Muhammad Safdar, Yasmeen Junejo, Fahad A. Alshanbari

**Affiliations:** 1 Department of Genetics & Bioinformatics, Faculty of Animal Production and Technology, Cholistan University of Veterinary and Animal Sciences, Bahawalpur 63100, Pakistan; tanveernasir408@gmail.com; 2 College of Animal Science and Technology, Nanjing Agricultural University, Nanjing 210095, China; tariq@stu.njau.edu.cn; 3 Department of Clinical Sciences, College of Veterinary Medicine, Qassim University, Buraidah 51452, Saudi Arabia; atieh@qu.edu.sa; 4 Department of Breeding & Genetics, Faculty of Animal Production and Technology, Cholistan University of Veterinary and Animal Sciences, Bahawalpur 63100, Pakistan; 5 Institute of Molecular Biology and Biotechnology, Foundation for Research and Technology-Hellas, 70013 Heraklion, Crete, Greece; 6 Department of Physiology & Biochemistry, Faculty of Biosciences, Cholistan University of Veterinary and Animal Sciences, Bahawalpur 63100, Pakistan; yasmeen@cuvas.edu.pk; 7 Department of Medical Biosciences, College of Veterinary Medicine, Qassim University, Buraidah 51452, Saudi Arabia

**Keywords:** apoptosis, Equidae, evolutionary changes, functional mutations, gene duplication, subcellular localization, TGF-β superfamily, tissue-specific expression, transforming growth factor beta

## Abstract

The transforming growth factor-beta (TGF-β) superfamily regulates diverse biological processes, including proliferation, differentiation, apoptosis, tissue remodeling, and reproductive signaling across metazoans. Here, we performed a genome-wide characterization of the TGF-β gene family in donkey (*Equus asinus*, ASM1607732v2) using comparative genomics and bioinformatics analyses, with horse (*Equus caballus*, EquCab3.0) as a reference to investigate evolutionary conservation and functional divergence. Genome assemblies and proteomes were retrieved from NCBI, and TGF-β genes were identified using BLASTp and HMMER searches (Pfam PF00019), followed by phylogenetic, conserved motif, synteny, Ka/Ks, mutation prediction, subcellular localization, and tissue-specific expression analyses. We identified 40 TGF-β genes in donkeys, exceeding the numbers reported in several mammals, suggesting possible lineage-specific expansion or differential gene retention within Equidae. Phylogenetic and motif analyses demonstrated strong evolutionary conservation across the two principal clades (TGF-β-like and BMP-like). Four segmental duplications were identified, with Ka/Ks ratios ranging from 0.28 to 0.43, indicating strong purifying selection on duplicated genes. Synteny analysis revealed extensive collinearity with the horse genome, supporting conserved equid genomic architecture. Comparative sequence analysis identified 160 amino acid variants, including 11 predicted deleterious mutations in key genes (*GDF6*, *GDF9*, *GDF10*, *BMP15*, and *RGMA*), suggesting potential functional divergence associated with reproductive and developmental pathways. Importantly, transcriptomic validation using publicly available donkey RNA-seq tissue expression data (NCBI BioProject: PRJNA1017964) revealed distinct tissue-specific expression patterns, with reproductive tissues (ovary and uterus) displaying enriched expression of TGF-β/BMP signaling components, particularly *TGFBR1*, *TGFBR2*, *TGFB1*, *BMP2*, *BMP4*, and *BMP7*, while canonical fecundity genes (GDF9 and BMP15) exhibited ovary-associated expression. This receptor-dominant signaling profile may have a coordinated TGF-β regulatory network underlying folliculogenesis, reproductive tissue remodeling, and fertility-related processes in donkeys. Subcellular localization predictions showed that most proteins (22/40) were extracellularly localized, consistent with conserved signaling functions. Together, this study provides the first integrated genomic and tissue-expression atlas of the donkey TGF-β superfamily, offering new insights into equid-specific evolutionary conservation, reproductive signaling, and functional divergence.

## Simple Summary

Donkeys play a significant role in transport, milk generation, and rural living, but their genetic composition has not been as widely studied as that of horses. The transforming growth factor-beta family is an important gene family involved in growth, reproduction, tissue formation, embryonic development, and functions in most species. This study was able to determine and examine every member of this gene family in the donkey genome and compare them to horses and other mammals. We identified 40 highly conserved genes in the evolutionary process, yet we also discovered instances of gene duplication and a variety of genetic alterations, which can affect reproduction and bone formation. It was predicted that some of the mutations would impact biological functions that were crucial, e.g., follicle development and embryogenesis. The findings enhance the comprehension of the genetics of the donkey and offer precious information that can be used in breeding programs in the future, to enhance reproduction and development characteristics.

## 1. Introduction

The transforming growth factor-beta (TGF-β) superfamily encompasses a large group of signaling molecules that govern developmental and physiological processes across metazoans. Comprising three canonical isoforms (TGF-β1, TGF-β2, and TGF-β3), these factors form dimeric polypeptides that activate conserved signaling cascades [1,2]. TGF-β family members are evolutionarily conserved across invertebrates and vertebrates and control fundamental developmental events, including dorsoventral patterning, mesoderm induction, limb formation, neurogenesis, and skeletogenesis [3,4,5]. In mammals, TGF-β signaling coordinates both cellular and organismal functions, regulating proliferation, apoptosis, adhesion, migration, angiogenesis, wound healing, and fibrosis [6]. Reproductive tissues are particularly sensitive to TGF-β activity. In cattle, TGF-β isoforms modulate granulosa cell steroidogenesis and follicular maturation, whereas in horses, they facilitate trophoblast differentiation and successful implantation, underscoring their critical role in reproductive physiology [7,8].

TGF-β proteins exert their regulatory functions primarily through the canonical Smad pathway, interacting with transcription factors and coregulators to modulate gene expression [9]. They also influence noncoding RNA networks, including microRNAs (miRNAs) and long noncoding RNAs (lncRNAs), shaping epigenetic landscapes essential for cell fate determination [10]. The superfamily includes more than 30 genes in mammals, such as bone morphogenetic proteins (BMPs), growth differentiation factors (GDFs), activins, inhibins, and nodal proteins, which collectively support diverse developmental and physiological functions [6]. Functional specialization of TGF-β genes has been reported in several domestic species. In buffalo, GDF9 enhances oocyte competence during in vitro maturation [11]. In cattle, BMP1 regulates granulosa cell proliferation and apoptosis, while BMP4/Smad signaling promotes follicular growth and ovulation [12,13]. In horses, MSTN polymorphisms are linked to variation in muscle development and racing performance [14].

Genome-wide identification and comparative genomic analyses have been extensively applied in plants, livestock, birds, and other organisms to characterize gene families, assess genetic diversity, and understand functional variation across species [15,16,17,18,19,20]. These studies typically integrate sequence homology searches (BLASTp), profile-based domain detection (HMMER), phylogenetic reconstruction, synteny analysis, selection pressure estimation (Ka/Ks), motif and domain characterization, and in silico functional prediction tools to provide evolutionary and biological insights into gene family dynamics [21]. Despite the economic importance of donkeys (*Equus asinus*) in transport, milk production, and rural livelihoods, the donkey genome remains comparatively understudied relative to the horse (*Equus caballus*) [22]. To date, no genome-wide characterization of the TGF-β superfamily has been reported in *Equus asinus*, leaving major gaps in our understanding of equid-specific evolutionary diversification, gene duplications, and functional variants associated with reproduction, growth, and developmental adaptation. Furthermore, tissue-level expression evidence supporting the biological relevance of TGF-β family genes in donkey reproductive and non-reproductive tissues remains unexplored. Integrating comparative genomics with transcriptomic validation may therefore provide deeper insights into tissue-specific signaling patterns and the potential functional significance of candidate genes within Equidae.

The objectives of this study were to (i) identify and classify all TGF-β family members in the donkey genome, (ii) perform phylogenetic, structural, synteny, and selection-pressure analyses in comparison with horse and other mammals, (iii) predict functional mutations and subcellular localizations to identify candidate variants with potential impacts on reproductive and developmental pathways, and (iv) evaluate tissue-specific expression profiles of key TGF-β family genes using publicly available donkey RNA-seq transcriptomic datasets (NCBI BioProject: PRJNA1017964) to provide functional support for reproductive and physiological relevance. All analyses were conducted using established comparative genomics and bioinformatics pipelines, including BLASTp and HMMER for gene identification, MEGA7 for phylogeny, MCScanX for synteny and duplication analyses, MEME for conserved motif identification, DnaSP for Ka/Ks estimation, multiple mutation-prediction tools (SIFT, PROVEAN, PolyPhen-2, among others), and WoLF PSORT for subcellular localization. Transcript abundance patterns were further examined across donkey tissues to assess tissue-specific enrichment of reproductive signaling components. This study provides the first integrated genomic and transcriptomic characterization of the donkey TGF-β superfamily and identifies candidate genes potentially involved in reproductive signaling, developmental regulation, and equid-specific evolutionary adaptation.

## 2. Materials and Methods

### 2.1. Data Retrieval

Whole-genome assemblies, proteomes, and GFF3 annotation data for donkey (*Equus asinus*, ASM1607732v2 Equus, RefSeq accession: GCF_016077325.2), horse (*Equus caballus*, EquCab3.0, RefSeq accession: GCF_002863925.1), cattle (*Bos taurus*, ARS-UCD1.2, RefSeq accession: GCF_002263795.1), sheep (*Ovis aries*, Oar_rambouillet_v1.0, RefSeq accession: GCF_002742125.1), goat (*Capra hircus*, ARS1.2, RefSeq accession: GCF_001704415.2), and human (*Homo sapiens*, GRCh38.p12, RefSeq accession: GCF_000001405.38) were retrieved on 12 March 2026 from the NCBI Genome database [23]. Non-redundant reference protein sequences for TGF-β family members from horse, cattle, sheep, goat, and human were obtained from UniProt [24]. All genome assembly accession numbers are provided in consistent NCBI format: donkey (ASM1607732v2), horse (EquCab3.0), cattle (ARS-UCD1.2), sheep (Oar_rambouillet_v1.0), goat (ARS1), and human (GRCh38.p12). The following protein accession IDs of the donkey TGF-β superfamily members were analyzed in this study: XP_014683912.1, XP_014705358.1, XP_014696363.1, XP_014705576.1, XP_044624318.1, XP_014699053.2, XP_014683835.1, XP_014702629.1, XP_044635082.2, XP_044610020.1, XP_044604909.1, XP_044629219.2, XP_014697014.3, XP_014706486.2, XP_014706241.1, XP_014703240.3, XP_044627936.1, XP_014712290.3, XP_014697015.2, XP_014712579.2, XP_044629495.2, XP_044622730.1, XP_014684231.2, XP_014691802.1, XP_014720199.1, XP_014707242.1, XP_044629204.2, XP_014687467.1, XP_014692224.1, XP_014682648.1, XP_044626354.2, XP_044626372.2, XP_044615807.1, XP_044622279.1, XP_014685900.2, XP_014700816.2, XP_070357667.1, XP_014697997.1, XP_044635996.1, XP_014702788.1, XP_014714415.1.

### 2.2. Identification of TGF-β Genes in Donkey

TGF-β protein isoforms in the donkey genome were identified using BLASTp v2.17.0 [25] and HMMER v3.4 [26]. BLASTp searches were performed with UniProt query sequences against the donkey proteome using an e-value threshold of ≤1 × 10^−5^, the BLOSUM62 scoring matrix, word size of 6, gap open cost of 11, gap extension cost of 1, and conditional composition score adjustment enabled. HMMER searches used the Pfam TGF-β domain profile (PF00019) [27] with a cutoff e-value of ≤1 × 10^−5^. The redundant sequences were removed using CD-HIT v4.8.1 with a 0.95 identity threshold. Candidate sequences were validated for TGF-β family domains using SMART (v8.0) and the NCBI Conserved Domain Database (CDD, accessed on 12 January 2026).

### 2.3. Characterization of Physicochemical Properties

Physicochemical properties of donkey TGF-β proteins—including amino acid length, molecular weight (MW), theoretical isoelectric point (pI), instability index (II), aliphatic index (AI), and GRAVY (grand average of hydropathicity)—were calculated using ProtParam (https://web.expasy.org/protparam/, accessed 12 March 2026). All FASTA sequences were manually curated prior to analysis.

### 2.4. Multiple Sequence Alignment

Donkey TGF-β protein sequences and comparative orthologous sequences were aligned using the multiple alignment show tool with MEGA7 [28] and default parameters (gap open penalty = 10; gap extension penalty = 0.2). Alignments were manually inspected to identify conserved regions, insertions, deletions, and amino acid substitutions.

### 2.5. Structural Analyses (Motifs, Gene Structure, Domains)

Conserved motifs were identified using MEME Suite v5.5.5 [29] with the following parameters: zero or one occurrence per sequence, a maximum of 10 motifs, and motif widths ranging from 6 to 50 amino acids. Gene structures were analyzed using the Gene Structure Display Server (GSDS) [30] by aligning genomic sequences with their corresponding coding sequences. Protein domain architectures were annotated using Pfam [27] and the NCBI Conserved Domain Database (CDD). Gene structures were visualized using TBtools (v2.0) [31].

### 2.6. Phylogenetic Analysis

Protein sequences from donkey, horse, cattle, sheep, goat, and human were aligned with MEGA7 [20]. A neighbor-joining (NJ) phylogenetic tree in Newick format was constructed using MEGA7 [28] (https://megasoftware.net/, accessed 12 January 2026) with the Poisson substitution model (chosen as the default model for neighbor-joining trees of protein sequences in MEGA7, providing robust distance estimation without assuming complex evolutionary rates), pairwise deletion for gaps/missing data, and 1000 bootstrap replicates to assess node support.

### 2.7. Synteny and Gene Duplication Analyses

Chromosomal positions of donkey TGF-β genes were extracted from the ASM1607732v2 genome annotations. MCScanX (version 1.1.11) [29] was used to identify collinear blocks and gene duplication events using the following parameters: a minimum of 5 collinear genes (a standard threshold in MCScanX to ensure detection of statistically robust syntenic blocks while minimizing spurious collinearity) and *E*-value ≤ 1 × 10^−10^. Donkey–horse synteny plots were visualized using TBtools v2.0 [31]. Homologous gene pairs were realigned using MUSCLE v3.8.31 to confirm duplication relationships.

### 2.8. Ka/Ks Calculation and Selection Pressure

Synonymous (Ks) and nonsynonymous (Ka) substitution rates for paralogous gene pairs were calculated using DnaSP (v6.12) [30] (http://www.ub.edu/dnasp/, accessed 10 September 2025) with multi-hit correction. Divergence times (T, in million years ago, MYA) were estimated using the equation

$$T=\frac{K_{s}}{2\lambda }$$

where λ = 1.26 × 10^−8^ substitutions per site per year [32].

### 2.9. SNP Detection and Functional Effect Prediction

Protein sequences of donkey and horse TGF-β genes were aligned using MEGA7 [27], and amino acid variants were visualized using multiple sequence alignment with MEGA7. Functional effects of nonsynonymous mutations were predicted using SIFT (version 6.2.1) [33], PROVEAN (release 1.1.5) [34], PhD-SNP (2010 release) [35], I-Mutant 3.0 (https://busca.biocomp.unibo.it/betaware/, accessed on 12 January 2026), PolyPhen-2 (http://genetics.bwh.harvard.edu/pph2/, accessed on 12 January 2026), and MUpro 9 https://mupro.proteomics.ics.uci.edu/, accessed on 12 January 2026) and others (https://snps.biofold.org/meta-snp/, accessed on 12 January 2026). Default parameters were used unless otherwise indicated. A mutation was classified as deleterious only by consensus (predicted deleterious by at least four of the six tools. Individual tool outputs are provided in Supplementary Tables S1–S40.

### 2.10. Subcellular Localization Prediction

Subcellular localization of donkey TGF-β proteins was predicted with WoLF PSORT online server (https://wolfpsort.hgc.jp/), accessed on 12 January 2026 [36] using the animal model and k-nearest neighbors (k = 10), the default and recommended setting for animal proteins to maximize prediction accuracy and specificity. Therefore, predicted compartments included extracellular, nuclear, plasma membrane, cytoplasmic, and mitochondrial localizations.

### 2.11. Tissue-Specific Expression Analysis of TGF-β Genes

To provide transcriptomic support for the identified TGF-β family genes, publicly available donkey RNA-seq expression data were retrieved from the NCBI Sequence Read Archive (SRA) BioProject PRJNA1017964. Expression data were obtained from the donkey transcriptome resource generated by Wang et al. [37] (NCBI BioProject: PRJNA1017964). Tissue-specific transcript abundance data from multiple donkey tissues, including ovary, uterus, pineal gland, spleen, blood, quadriceps femoris, and longissimus dorsi, were used to examine expression patterns of TGF-β family members. Gene expression levels were evaluated as transcripts per million (TPM) to assess tissue-specific enrichment and biological relevance of candidate genes involved in reproductive and developmental signaling pathways.

### 2.12. Protein–Protein Interaction and KEGG Pathway Enrichment Analysis

To examine functional interactions of the identified TGF-β superfamily proteins, a protein–protein interaction (PPI) network was drawn with the help of the STRING database (version 11.5). The protein sequences were mapped to their orthologs, and interaction data were retrieved using experimental evidence, curated databases, co-expression, gene neighborhood, gene fusion, and text mining. A confidence score threshold of ≥0.4 was applied to filter interactions. The resulting interaction network was directly visualised with the STRING web interface, with nodes denoting proteins and edges denoting the predicted functional interactions.

To conduct functional characterization, the STRING enrichment module was used to conduct KEGG pathway enrichment analysis. False discovery rate (FDR)-adjusted *p*-values were used to assess statistical significance. The top enriched pathways were selected based on both statistical significance and biological relevance. The results of the enrichment were plotted in Python (version 3.13) with the Matplotlib (3.10.x) library, with the value of −log10(FDR) indicating the significance of a given pathway and the bubble size denoting the number of genes that participated in this pathway.

## 3. Results

### 3.1. Genome-Wide Identification of Donkey TGF-β Genes

**Figure 1:**
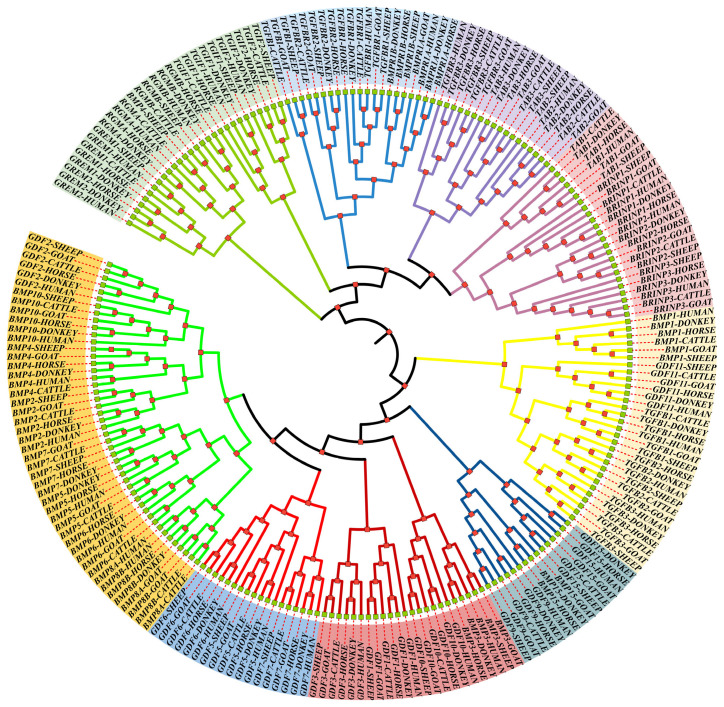
Genome-wide identification and phylogenetic classification of TGF-β genes across six mammals. The bar graph shows the total number of TGF-β genes identified in each species by BLASTp and HMMER searches. The neighbor-joining phylogenetic tree (Poisson model, 1000 bootstrap replicates) classifies donkey TGF-β genes into TGF-β-like and BMP-like clades. Bootstrap support values > 70%. Domain composition of each gene was validated using SMART and NCBI-CDD.

BLAST and HMMER analyses across six mammals (donkey, horse, cattle, sheep, goat, human) identified 205 non-redundant TGF-β-encoded protein sequences. In donkey (*Equus asinus*, ASM1607732v2), 40 TGF-β genes were identified, exceeding the counts observed in several mammals. This observation may reflect lineage-specific expansion or differential gene retention within Equidae, although additional comparative genomic analyses are required to confirm this evolutionary scenario, which may have contributed to the diversification of growth and reproductive traits in donkeys. Domain annotation using SMART and NCBI-CDD confirmed the presence of canonical TGF-β domains in all candidates, ensuring a high-confidence gene set (Figure 1). This expansion in donkey may be related to other mammals, highlighting potential Equidae-specific evolutionary adaptations in TGF-β signaling.

### 3.2. Evolutionary Analysis

Phylogenetic analysis, supported by bootstrap values greater than 70%, separated the sequences into two primary clades: TGF-β-like and BMP-like. The TGF-β-like clade was further divided into five subgroups (NODAL, GDF10/BMP3, GDF11/MSTN, TGF-β, and INHIBIN), while the BMP-like clade comprised BMP2/4/6, GDF2, GDF5/6/7, BMP5/6/7/8A/8B, GDF1/3/9/15, and BMP15 (Figure 1). Donkey genes exhibited higher sequence similarity to horse orthologs than to ruminants, consistent with the close evolutionary relationship within Equidae. Notably, expansion in the TGF-β-like subgroups may support species-specific regulatory adaptations in growth and reproductive pathways.

**Figure 2:**
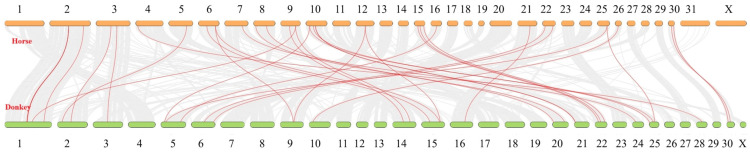
Chromosomal distribution and collinearity of donkey TGF-β genes with the horse genome. Donkey genes are mapped to 14 chromosomes (colored by subfamily). Orthologous relationships are shown as colored lines. Segmental duplication pairs are highlighted in red. TGF-β-like and BMP-like subfamilies are represented using distinct color gradients.

Four segmental duplications were identified. Synteny analysis revealed high collinearity with the horse genome, reflecting conserved equid genomic architecture despite species divergence. Donkey TGF-β genes were mapped to 14 chromosomes, whereas horse genes spanned 16, predominantly at distal chromosomal ends (Figure 2, Table 1). Ka/Ks ratios of 0.28–0.43 indicated strong purifying selection acting on duplicated genes, suggesting that these paralogs have been functionally conserved rather than nonfunctionalized.

### 3.3. Structural and Physicochemical Characterization

**Figure 3:**
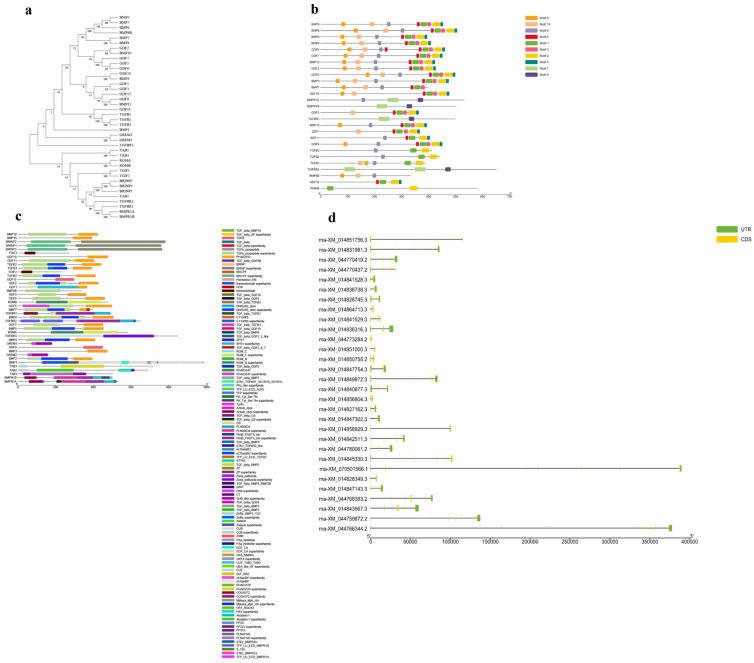
Structural and motif analysis of the 40 donkey TGF-β genes. (**a**) Phylogenetic tree of donkey TGF-β proteins (same as in Figure 1). (**b**) Conserved MEME motifs (motifs 1 and 2 correspond to the canonical TGF-β domain). (**c**) Protein domain architecture identified by NCBI-CDD and Pfam (TGFb_propeptide domains highlighted). (**d**) Exon–intron organization and 5′/3′ UTRs visualized with GSDS. Color coding in panels (**b**,**c**) represents individual motifs and domains, respectively.

Integration of phylogenetic relationships, motif architecture, conserved domains, and exon–intron organization revealed structural diversity among donkey TGF-β genes, with seven conserved MEME motifs identified (Figure 3a–d). Motifs 1 and 2 corresponded to the canonical TGF-β domains (Figure 3b; Table 2), whereas TGFb_propeptide domains were identified in selected genes (Figure 3c). Variation in exon–intron organization and the diversity of 5′ and 3′ untranslated regions (UTRs) suggest regulatory complexity that may contribute to differential gene expression across tissues and developmental stages (Figure 3d). Furthermore, as shown in Table 3, the predicted molecular weights ranged from 34.5 to 56.2 kDa, while the theoretical isoelectric points (pI) ranged from 4.95 to 10.35. Most proteins were basic, except for TAB1, TGFBR2, TGFBR3, BMP1, and BMP10, which exhibited pI values below 7. High aliphatic index (AI) values (>60) suggested good thermostability, whereas negative GRAVY scores indicated that the analyzed TGF-β family proteins are predominantly hydrophilic (Table 2). Collectively, the coexistence of conserved structural features and lineage-specific variations suggests that the donkey TGF-β superfamily has evolved under strong evolutionary constraints while maintaining regulatory flexibility, thereby preserving essential biological functions and facilitating potential functional divergence of TGF-β signaling in donkeys.

### 3.4. Mutation Analysis

Comparative sequence analysis uncovered 160 amino acid variants, including 11 predicted deleterious mutations in GDF6 (3), GDF9 (2), GDF10 (4), BMP15 (1), and RGMA (1), highlighting potential functional divergence affecting reproductive and developmental pathways. Protein sequences of donkey and horse TGF-β genes were aligned using ClustalW (v2.1) and visualized in BioEdit (v7.2.5). Functional effects of nonsynonymous mutations were predicted using SIFT (version 6.2.1), PROVEAN (release 1.1.5), PolyPhen-2 (HumVar model, 2013 release), I-Mutant 3.0 (2010 release), Phd-SNP (2010 release), and MUpo (2006 release). Consensus results are presented in Table 4 and Supplementary Tables S1–S40.

### 3.5. Functional Prediction

**Figure 4:**
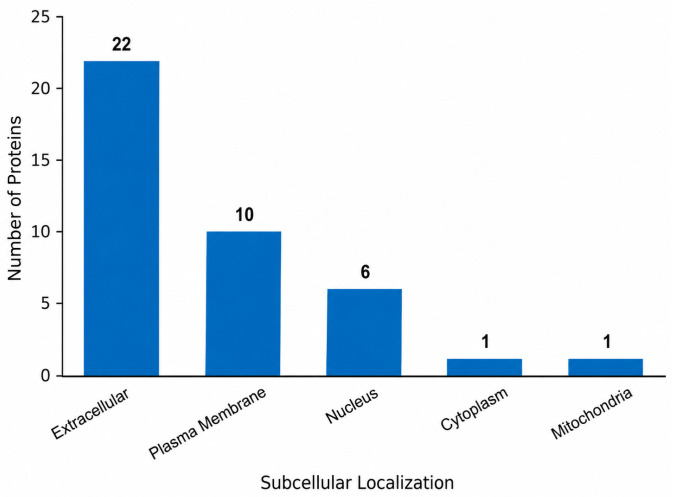
Distribution of predicted subcellular localization of donkey TGF-β family proteins using WoLF PSORT. The majority of TGF-β proteins were predicted to localize to the extracellular space, followed by the plasma membrane and nucleus, consistent with their conserved signaling and receptor-mediated functions.

Subcellular localization was predicted using WoLF PSORT, with the majority localized extracellularly, supporting signaling-related functions, with additional nuclear, plasma membrane, cytoplasmic, and mitochondrial distributions. Together, these computational predictions suggest that the identified variants and predicted localizations may be associated with biological functions related to reproduction, growth, and embryogenesis, although experimental validation is required (Figure 4).

### 3.6. Tissue-Specific Expression Profiling of Donkey TGF-β Family Genes

To assess the potential functional relevance of donkey TGF-β family genes, tissue-specific transcript abundance profiles were examined using publicly available donkey RNA-seq data (NCBI BioProject: PRJNA1017964). Expression analysis across the ovary, uterus, pineal gland, spleen, blood, quadriceps femoris, and longissimus dorsi revealed marked tissue-specific variation in the transcriptional activity of TGF-β signaling components (Table 5). Reproductive tissues, particularly the ovary and uterus, exhibited enriched expression of several TGF-β/BMP pathway genes. In the ovary, TGFBR2 displayed the highest transcript abundance, followed by TGFBR1, TGFB1, BMP2, BMP7, and BMP4, indicating active receptor-mediated signaling. Canonical fertility-associated genes, GDF9 and BMP15, also showed ovary-associated expression, supporting their conserved roles in folliculogenesis, oocyte maturation, and ovulation. Notably, the predominance of receptor transcripts over ligand expression suggests a coordinated and potentially receptor-dominant regulatory environment in donkey ovarian tissue. Similarly, the uterus exhibited strong expression of TGFBR2, BMP4, GDF11, TGFBR1, TGFB2, and BMP7, highlighting an active signaling landscape potentially associated with tissue remodeling and reproductive tract function. Expression of TGF-β signaling genes in the pineal gland further suggested a possible neuroendocrine contribution to reproductive physiology through endocrine signaling pathways.

In contrast, non-reproductive tissues, including spleen, blood, and skeletal muscles (quadriceps femoris and longissimus dorsi), displayed broader but distinct expression profiles. High expression of TGFBR2, TGFB1, BMPR1A, and BMP4 in spleen and muscle tissues supports conserved roles in immune modulation, tissue maintenance, and developmental signaling. Blood tissue showed comparatively restricted expression, dominated by TGFBR1, TGFBR2, and BMP2, indicating systemic signaling responsiveness. Overall, these findings demonstrate tissue-specific enrichment of TGF-β family genes in donkeys and provide transcriptomic support for their involvement in reproductive and developmental processes. The coordinated expression of ligands and receptors in reproductive tissues reinforces the functional significance of TGF-β signaling in equid fertility biology.

### 3.7. Integrated PPI and KEGG Enrichment Analysis

**Figure 5:**
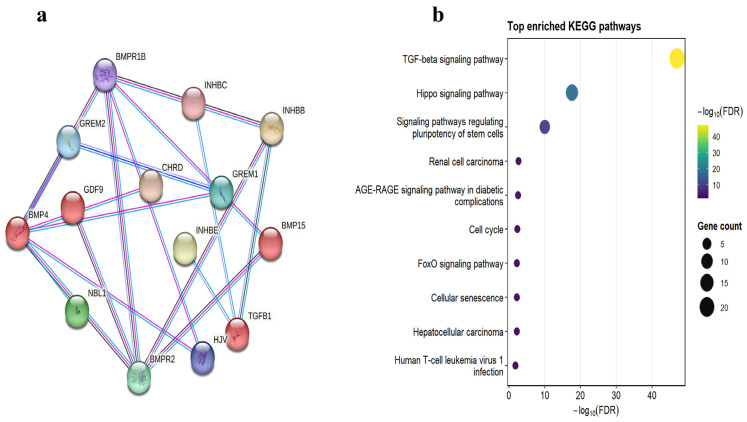
Integrated PPI and KEGG pathway enrichment analysis of TGF-β genes in donkeys. (**a**) Protein–protein interaction network generated using STRING. Nodes represent proteins and edges indicate functional associations. (**b**) KEGG pathway enrichment analysis. The x-axis shows −log10(FDR), and bubble size represents gene count. Key enriched pathways include TGF-β signaling, Hippo signaling, and stem cell pluripotency.

In order to further clarify the functional interaction and biological role of the identified TGF-β superfamily genes, a combined analysis based on protein–protein interaction (PPI) network and KEGG pathway enrichment was conducted (Figure 5).

STRING-based PPI network predicted that the interaction landscape was highly interconnected, demonstrating that the components of TGF-β signaling are strongly functionally connected (Figure 5a). A number of proteins, such as TGFB1, BMPR1B, BMP4, BMP2, and GDF9, were identified as predicted hub nodes with high connectivity. In particular, TGFB1 was predicted to interact strongly with BMP ligands and receptor proteins (BMPR2 and BMPR1B), highlighting its central role in canonical TGF-β signaling pathways. The BMP and GDF subfamilies (BMP4, BMP15, GDF9, and GDF6) were present in the network as highly linked clusters, indicating some coordinated role in developmental and reproductive events. The interaction between GDF9 and BMP15, as observed, also supports the already established roles of the two in folliculogenesis and oocyte maturation, in line with the ovary-enriched expression patterns of the two in this study. Moreover, the regulatory factors (GREM1, GREM2, and CHRD) were incorporated into the network, which means that they are extracellular antagonists that control BMP signaling activity. Likewise, the core signaling proteins were also linked to inhibin subunits (INHBA, INHBB, and INHBC), which further highlights their role in the regulation of reproductive hormones.

Enrichment analysis of KEGG pathways (Figure 5b) revealed that there is significant enrichment in pathways related to TGF-β signaling, Hippo signaling, and stem cell pluripotency in addition to other regulatory processes that include cell cycle, FoxO signaling, and cellular senescence. The identified gene family is functionally relevant as these pathways are mostly engaged in cell differentiation, proliferation, and developmental regulation. Even though some disease-related pathways were also enriched, they probably represent overlapping molecular signaling pathways, as in most studies of pathway enrichment of conserved signaling networks. In general, the integrated PPI and KEGG results show that the donkey TGF-β gene family is an integrated and coordinated signaling network, with critical roles in developmental control, cellular signaling, and reproductive functions.

## 4. Discussion

Advances in high-throughput genome sequencing have enabled genome-wide identification of functional gene families and genetic variants across diverse organisms, including livestock, plants, avian species, and pathogenic bacteria [37,38,39]. In livestock, candidate gene studies increasingly integrate genomic resources with transcriptomic evidence to identify functional genes associated with economically important traits, such as disease resistance, reproductive efficiency, and environmental adaptation [40,41]. Comparative genomics between donkeys and horses provides a valuable framework for uncovering species-specific adaptations and the genetic basis of economically important traits [42,43].

In this study, a total of 40 TGF-β genes were identified in the donkey genome (*Equus asinus*, ASM1607732v2) and classified into TGF-β-like and BMP-like groups. The TGF-β-like group was further divided into five subgroups: NODAL, GDF10/BMP3, GDF11/MSTN, TGF-β, and INHIBIN, whereas the BMP-like group comprised BMP2/4/6, GDF2, GDF5/6/7, BMP5/6/7/8A/8B, GDF1/3/9/15, and BMP15 (Figure 1). This phylogenetic organization is consistent with previous studies demonstrating strong evolutionary conservation of the TGF-β superfamily across vertebrates [44,45,46]. However, the number of TGF-β family members identified in donkeys was slightly higher than that reported in several mammals [47,48], including the closely related horse, suggesting potential lineage-specific expansion or differential retention of duplicated genes. Although additional evolutionary analyses are needed to determine whether these differences represent true biological diversification or annotation variation, the observed expansion may reflect species-specific functional requirements within Equidae.

Structural analyses identified seven conserved motifs, including TGF-β and cystine-knot cytokine domains [45] (Table 1, Figure 3), which are essential for ligand–receptor interactions and downstream signaling [48,49]. Four segmental duplications (GDF2/BMP10, GDF1/GDF3, GDF5/GDF6, and GREM1/GREM2) were identified, with Ka/Ks ratios ranging from 0.28 to 0.43, indicating strong purifying selection (Table 3). High synteny with the horse genome (Figure 2) further supports the conserved genomic architecture of equids, consistent with previous comparative studies [42,43]. Although genomic resources for non-equid perissodactyls such as rhinoceros and tapir remain limited [40,50], the observed genomic conservation appears characteristic of Equidae. Comparative sequence analysis revealed 160 amino acid variants between donkey and horse TGF-β orthologs, including 11 predicted deleterious substitutions in GDF6, GDF9, GDF10, BMP15, and RGMA (Table 4). Notably, some of these variants occurred in genes with established reproductive functions. GDF9 and BMP15 are well-recognized regulators of folliculogenesis, oocyte maturation, and ovulation in mammals [51,52], while GDF6 and GDF10 contribute to developmental signaling and skeletal formation [53,54]. Although the phenotypic effects of these variants remain unresolved in donkeys, their occurrence in functionally relevant genes suggests potential contributions to species-specific reproductive and developmental traits that warrant future experimental investigation.

Importantly, transcriptomic validation using publicly available donkey RNA-seq data (NCBI BioProject: PRJNA1017964) provided additional functional support for the biological relevance of TGF-β family genes. Tissue-specific expression profiling demonstrated that reproductive tissues, particularly the ovary and uterus, exhibited enriched expression of several pathway components, including TGFBR1, TGFBR2, TGFB1, BMP2, BMP4, and BMP7. Canonical reproductive genes such as GDF9 and BMP15 showed ovary-associated expression, consistent with their conserved roles in oocyte–granulosa cell communication and follicular development [7,55]. Interestingly, receptor genes (TGFBR1 and TGFBR2) displayed stronger transcript abundance than several canonical ligands, suggesting that reproductive regulation in donkeys may involve a receptor-dominant TGF-β signaling environment rather than dependence on a limited number of ovary-specific factors alone. Such coordinated expression of ligands and receptors may contribute to follicular maturation, reproductive tissue remodeling, and fertility-related physiology. While the expression patterns in non-reproductive tissues further supported the pleiotropic nature of the TGF-β superfamily. Spleen and muscle tissues showed broader expression of signaling components, including TGFB1, BMP4, BMPR1A, and TGFBR2, consistent with established functions in immune modulation, tissue maintenance, and developmental regulation [56,57]. Likewise, the presence of TGF-β signaling genes in the pineal gland suggests possible neuroendocrine contributions to reproductive physiology, potentially linking endocrine timing with reproductive function in equids. Together, these findings indicate that the donkey TGF-β superfamily exhibits tissue-specific transcriptional specialization while retaining broad physiological functionality.

Subcellular localization predictions suggested that most proteins (22/40) are extracellularly localized (Figure 4), consistent with their established functions as secreted signaling molecules within the TGF-β superfamily [7,55]. Protein–protein interaction analyses further identified hub genes such as TGFB1, BMP4, and GDF9 (Figure 5), mirroring interaction patterns reported in cattle, sheep, and humans [58]. The observed interactions between GDF9 and BMP15 are particularly noteworthy, given their conserved cooperative roles in follicular maturation and female fertility [59,60]. Overall, this study provides the first integrated genomic and transcriptomic characterization of the donkey TGF-β superfamily, revealing conserved evolutionary architecture, duplicated genes under purifying selection, candidate deleterious variants, and tissue-specific expression patterns associated with reproductive and developmental biology. These findings broaden our understanding of equid molecular evolution and provide a foundation for future functional genomics and breeding applications.

### Limitations and Future Directions

Although this study provides robust in silico evidence of donkey-specific duplications and deleterious mutations, the findings are based on computational predictions. Functional validation through targeted gene expression analysis, protein interaction assays, CRISPR editing, and phenotypic association studies is required to confirm biological relevance. Data for non-equid perissodactyls remain scarce, limiting broader evolutionary comparisons. Future functional and breeding studies building on this TGF-β atlas will help clarify the role of these variants in donkey reproduction, growth, and resilience, ultimately supporting marker-assisted selection programs. Furthermore, the predicted protein–protein interactions and pathway enrichments are based on database-derived computational models and should not be interpreted as direct experimental evidence in donkey.

## 5. Conclusions

This study presents the first genome-wide characterization of the TGF-β superfamily in donkey (*Equus asinus*), identifying 40 TGF-β family genes with strong evolutionary conservation, lineage-specific expansion, and four segmental duplications under purifying selection (Ka/Ks = 0.28–0.43). Comparative analyses identified 11 predicted deleterious variants in key genes (GDF6, GDF9, GDF10, BMP15, and RGMA), suggesting potential functional divergence related to reproductive and developmental processes. Transcriptomic validation using publicly available donkey RNA-seq data further revealed tissue-specific enrichment of TGF-β signaling genes, particularly in reproductive tissues (ovary and uterus), supporting their potential roles in folliculogenesis, reproductive tissue remodeling, and fertility-related signaling. Subcellular localization analysis indicated that most proteins are extracellular, consistent with conserved signaling functions. Finally, this study provides the first integrated genomic and transcriptomic resource for the donkey TGF-β superfamily and offers a valuable foundation for future functional genomics, reproductive biology, and breeding applications in equids.

## Figures and Tables

**Table 1:** Segmental duplication events in donkey TGF-β genes, with Ka/Ks ratios.

Gene Pairs	Chromosome	Duplication	Ka	Ks	Ka/Ks	MYA
GDF2/BMP10	ch2/12	SD	0.52	1.90	0.28	146.2
GDF1/GDF3	ch10/22	SD	0.66	1.54	0.43	118.5
GDF5/GDF6	ch15/12	SD	0.42	1.06	0.40	81.5
GREM1/GREM2	ch2/30	SD	0.36	0.93	0.39	71.5

SD (segmental duplication); Ka (non-synonymous substitutions); Ks (synonymous substitutions); MYA (Million Years Ago).

**Table 2:** The Conserved MEME motifs in donkey TGF-β proteins.

Motif	Sequence	Length	Pfam Domain
**1**	WIIAPKGYEAYYCEGECPFPL	21	TGF-beta family profile Transforming growth factor beta-like domain Cystine-knot cytokines TGF-beta family signature
**2**	PKPCCVPTKLSPISILYIDENNNVVLKKY	29	TGF-beta-like domain TGF-beta family profile Cystine-knot cytokines
**3**	KNACRRHPLYVSFKDLGWDD	20	TGF-beta family profile Cystine-knot cytokines
**4**	ASHLNPTNHAIIQTLVHSMNP	21	TGF-beta family profile Consensus disorder prediction
**5**	WLTFDVTAAVRRWLLNRQKNLGLRLSV	27	TGF-beta propeptide
**6**	ETEIYQTVLLRHENILGFIAADIKGTGSWTQLWLITDYHENGSLYDYLK	49	TGF-beta receptor type I&II Protein kinase domain profile Phosphorylase Kinase; domain 1 Protein kinase-like (PK-like)
**7**	AHRDLKSKNILVKKNGTCCIADLGLAVKFDSDTNEVDIPPNTRVGTKRYM	50	TGF-beta receptor type I&II Protein kinase domain profile Transferase (Phosphotransferase) domain 1 Protein kinase domain, Protein kinase-like (PK-like)

**Table 3:** Physicochemical properties of donkey TGF-β proteins.

Sr#	Gene	Chr.	Exon Count	A.A.	MW (Da)	pI	II	Al	GRAVY
**1**	TGFB1	26	7	391	44.07	8.83	46.29	89.77	−0.255
**2**	TGFB2	30	8	442	50.52	8.74	53.00	80.52	−0.396
**3**	TGFB3	7	7	412	47.17	8.13	48.59	82.06	−0.499
**4**	TAB1	4	11	504	54.60	5.31	45.98	82.44	−0.339
**5**	TAB2	1	9	693	76.51	8.83	58.96	63.74	−0.750
**6**	TAB3	X	13	717	79.04	8.81	78.93	57.81	−0.836
**7**	TGIF1	7	5	272	29.62	7.64	60.43	72.13	−0.489
**8**	TGIF2	15	5	237	25.80	7.77	58.88	84.35	−0.488
**9**	TGFBR1	10	11	499	55.53	7.51	42.82	90.90	−0.083
**10**	TGFBR2	21	10	652	73.87	5.44	44.56	78.16	−0.351
**11**	TGFBR3	16	17	850	93.18	5.74	48.53	81.13	−0.248
**12**	GDF1	10	2	368	39.04	10.79	71.84	90.87	−0.050
**13**	GDF2	2	2	427	47.39	6.34	50.53	74.89	−0.437
**14**	GDF3	22	2	364	40.76	7.11	52.09	97.25	−0.087
**15**	GDF5	15	2	499	55.28	9.87	46.43	71.02	−0.617
**16**	GDF6	12	2	461	51.41	9.19	65.48	69.65	−0.615
**17**	GDF7	6	2	452	46.46	9.29	55.16	79.51	−0.118
**18**	GDF9	9	2	451	50.71	8.92	57.08	78.96	−0.366
**19**	GDF10	2	2	477	52.53	9.52	57.24	77.38	−0.456
**20**	GDF11	22	2	405	44.92	8.18	60.02	80.99	−0.303
**21**	GDF15	10	2	300	32.93	10.56	61.91	93.50	−0.270
**22**	BMP1	3	22	988	11.17	6.27	46.65	61.84	−0.609
**23**	BMP2	15	3	395	44.86	8.72	52.88	79.24	−0.470
**24**	BMP3	3	3	475	53.45	9.62	59.67	79.05	−0.519
**25**	BMP4	7	4	409	46.59	8.57	57.89	79.80	−0.532
**26**	BMP5	8	7	454	51.47	9.00	47.99	77.97	−0.439
**27**	BMP6	8	7	506	55.78	8.38	59.65	71.76	−0.468
**28**	BMP7	15	8	431	45.35	8.36	54.40	73.69	−0.513
**29**	BMP10	6	2	424	48.02	4.84	47.26	86.93	−0.349
**30**	BMP15	X	2	394	45.22	9.67	52.86	96.19	−0.309
**31**	BMP8A	5	8	402	36.57	8.79	66.99	79.97	−0.471
**32**	BMP8B	5	7	402	60.13	7.42	45.57	87.97	−0.178
**33a**	BMPR1A	2	13	532	57.24	8.23	51.15	84.04	−0.327
**33b**	BMPR1B	3	24	491	88.77	9.15	48.04	78.02	−0.471
**34**	BRINP1	10	8	761	88.91	8.45	50.39	85.71	−0.314
**35**	BRINP2	25	8	783	88.18	8.06	58.97	83.26	−0.335
**36**	BRINP3	30	10	766	63.28	9.69	66.14	66.05	−0.586
**37**	RGMA	2	4	455	51.98	8.35	45.50	74.46	−0.299
**38**	RGMB	9	9	480	53.34	8.21	45.25	72.94	−0.339
**39**	GREM1	2	2	184	20.63	9.46	62.67	60.98	−0.776
**40**	GREM2	30	3	168	19.25	9.36	55.23	77.68	−0.486

Molecular weight (MW), isoelectric point (pI), instability index (II), aliphatic index (AI).

**Table 4:** Amino acid variants in donkey TGF-β proteins relative to horse orthologs. The predicted functional effects of deleterious and neutral variants are indicated.

**TAB 1**										
**Mutations**	**MetaSNP**	**MUPro**	**PhD-SNP**	**PROVEN**	**PolyPhen-2**	**SIFT**	**SNAP**	**I-Mutant**	**PANTHER**	**Overall**
**S260A**	NE	DE	NE	NE	BE	NE	NE	DE	NE	SYN
**TAB 2**										
**S337N**	NE	DE	NE	NE	BE	NE	NE	DE	NE	SYN
**A348T**	NE	DE	NE	NE	BE	NE	NE	DE	NE	SYN
**P353S**	NE	DE	NE	NE	BE	NE	DI	DE	NE	SYN
**A381S**	NE	DE	NE	NE	BE	NE	NE	DE	NE	SYN
**TGFBR2**										
**I36M**	NE	DE	NE	NE	BE	NA	DI	DE	NA	SYN
**H81R**	NE	DE	NE	NE	BE	NA	NE	DE	NA	SYN
**GDF1**										
**A259P**	NE	DE	NE	NE	BE	NE	NE	DE	NE	SYN
**L184P**	NE	DE	NE	NE	BE	NE	NE	DE	NE	SYN
**GDF2**										
**V135I**	NE	DE	NE	NE	BE	NE	NE	DE	NE	SYN
**T182A**	NE	DE	NE	NE	BE	NE	NE	DE	NE	SYN
**A308V**	NE	DE	NE	NE	BE	NE	NE	DE	NA	SYN
**GDF3**										
**A7V**	NE	DE	NE	NE	BE	NE	NE	DE	NA	SYN
**L13M**	NE	DE	NE	NE	BE	DI	NE	DE	NA	SYN
**GDF6**										
**R73P**	DI	DE	NE	NE	BE	NE	DI	DE	NE	NON-SYN
**L303P**	NE	DE	DI	NE	BE	NE	NE	DE	NA	NON-SYN
**V328A**	NE	DE	NE	NE	BE	NE	NE	DE	NA	NON-SYN
**GDF7**										
**E166D**	NE	DE	NE	NE	BE	NE	NE	DE	NE	SYN
**F167S**	NE	DE	NE	NE	BE	NE	NE	DE	DI	SYN
**P296A**	NE	DE	NE	NE	BE	NE	NE	DE	NA	SYN
**GDF9**										
**R87G**	DI	DE	DI	NE	BE	DI	DI	DE	NE	NON-SYN
**T310R**	NE	DE	NE	NE	BE	NE	NE	DE	NA	NON-SYN
**BMP15**										
**F17Y**	DI	DE	DI	NE	BE	NE	DI	DE	NE	NON-SYN
**Y150H**	NE	DE	NE	NE	BE	NE	NE	DE	DI	SYN
**BMP8B**										
**S6G**	NE	DE	NE	NE	BE	NE	NE	DE	NE	SYN
**V141I**	NE	DE	NE	NE	BE	NE	NE	DE	NE	SYN
**R188Q**	NE	DE	NE	NE	BE	NE	NE	DE	NE	SYN
**C257R**	NE	DE	NE	NE	BE	NE	NE	DE	NA	SYN
**P288S**	NE	DE	NE	NE	BE	NE	NE	DE	NA	SYN
**BMPR1A**										
**S5Y**	NE	DE	NE	NE	BE	NE	DI	DE	NA	SYN
**BMPR1B**										
**L462I**	NE	DE	NE	NE	BE	NE	NE	DE	NE	SYN
**BRINP2**										
**G367S**	NE	DE	NE	NE	BE	NE	NE	DE	NE	SYN
**RGMA**										
**P160S**	NE	DE	NE	NE	BE	DI	DI	DE	NA	NON-SYN
**GDF10**										
**R28Q**	DI	DE	DI	NE	BE	NE	DI	DE	NE	NON-SYN
**A225P**	NE	DE	NE	NE	BE	NE	NE	DE	NE	NON-SYN
**F347L**	NE	DE	NE	NE	BE	NE	NE	DE	NA	NON-SYN
**A348P**	NE	DE	DI	NE	BE	NE	NE	DE	NA	NON-SYN

NE: neutral effect; DE: deleterious effect; DI: disease-associated prediction; NA: not available; BE: benign; SYN: synonymous variant; NON-SYN: nonsynonymous variant; SNP: single nucleotide polymorphism. Bold gene names indicate gene-specific groups under which the corresponding amino acid variants are listed.

**Table 5:** Tissue-specific expression (TPM) profile of ovulation- and reproductive-related genes in donkey tissues.

Genes	Transcript IDs	Ovary	Uterus	Pineal Gland	Spleen	Blood	Quadriceps Femoris	Longissimus Dorsi
GDF9	XM_014856804.3	0.932	0.296	0.546	0.162	0	0.099	0
BMP15	XM_014827162.3	6.197	0.037	0	0.086	0	0.019	0.097
BMP4	XM_014864713.3	113.613	46.152	13.867	30.35	0.363	14.072	10.257
BMP7	XM_014831981.3	13.15	14.456	29.086	13.79	0.418	2.377	1.841
BMPR1B	XM_044766344.2	1.243	8.997	50.195	3.547	0.427	3.687	2.921
TGFB1	XM_044746676.2	25.771	11.338	13.867	30.35	0.363	19.954	26.793
GDF10	XM_014841529.3	1.366	0.229	5.02	11.017	0	5.1	2.696
TGFB2	XM_014849872.3	15.831	14.164	14.27	17.06	0.131	9.085	9.261
TGFB3	XM_014840877.3	19.474	9.276	5.317	11.76	0.785	7.789	5.245
TGFBR1	XM_044779147.2	30.227	18.186	23.611	7.482	43.372	8.348	9.644
TGFBR2	XM_044754085.2	137.097	67.166	67.163	39.59	61.603	53.212	35.848
BMPR1A	XM_044759872.2	28.712	25.709	15.172	22.89	1.75	19.576	24.253
RGMA	XM_014842511.3	11.219	7.949	3.406	11.23	0	22.316	13.281
BMP2	XM_044766204.2	17.381	16.869	10.472	5.585	40.005	5.471	5.418
GDF11	XM_014857093.3	13.268	27.791	18.021	3.403	1.011	3.623	8.502

TPM: Transcripts per million. NCBI SRA BioProject (PRJNA1017964) [37].

## Data Availability

The original contributions presented in this study are included in the article. Further inquiries can be directed to the corresponding authors.

## Supplementary Materials

The following supporting information can be downloaded at https://www.mdpi.com/article/10.3390/ani16132028/s1.

## Abbreviations

The following abbreviations are used in this manuscript:

AI	Aliphatic index
BLAST	Basic Local Alignment Search Tool
BMP	Bone morphogenetic protein
CDD	Conserved Domain Database
GDF	Growth differentiation factor
GFF3	General Feature Format version 3
GRAVY	Grand average of hydropathicity
HMM	Hidden Markov model
HMMER	Hidden Markov Model-based sequence analysis tool
II	Instability index
Ka	Nonsynonymous substitution rate
Ks	Synonymous substitution rate
lncRNA	Long noncoding RNA
MCScanX	Multiple Collinearity Scan toolkit
MEGA	Molecular Evolutionary Genetics Analysis
MEME	Multiple Expectation Maximization for Motif Elicitation
miRNA	MicroRNA
MSTN	Myostatin
MW	Molecular weight
MYA	Million years ago
NCBI	National Center for Biotechnology Information
NGS	Next-generation sequencing
NJ	Neighbor-joining
Pfam	Protein families database
pI	Isoelectric point
PROVEAN	Protein Variation Effect Analyzer
RGMA	Repulsive Guidance Molecule A
R-SMAD	Receptor-regulated SMAD
SIFT	Sorting Intolerant From Tolerant
SMART	Simple Modular Architecture Research Tool
SNP	Single nucleotide polymorphism
SMAD	Small Mothers Against Decapentaplegic
TBtools	Toolkit for Biologists
TGF-β	Transforming growth factor beta
TGFBR	Transforming growth factor beta receptor
UTR	Untranslated region
WoLF PSORT	Weighted k-nearest neighbor Protein Subcellular Localization Prediction Tool
