# Supplementary material for: Genome-Wide Characterization of the TGF-β Gene Family in Donkey (Equus asinus) Reveals Lineage-Specific Gene Duplications and Deleterious Mutations
Source: Animals (Basel). 2026 Jul 2;16(13):2028. doi: 10.3390/ani16132028 (PMC13359590; doi:10.3390/ani16132028)
Supplement: Supplementary file 1 [file animals-16-02028-s001.zip › animals-4299210-supplementary.pdf]

Table S1. Comparative amino acid analysis of donkey and horse TGFB1 gene

H.TGFB1<br>D.TGFB1	MPPSGLRLLPLLLPLLWLLVLTPGRPAAGLSTCKTIDMELVKRKRIEAIRGQILSKLRLA<br>MPPSGLRLLPLLLPLLWLLVLTPGRPAAGLSTCKTIDMELVKRKRIEAIRGQILSKLRLA	1<br>60
H.TGFB1<br>D.TGFB1	SPPSQGEVPPGPLPEAVLALYNSTRAQVAGESAETEPEPEADYYAKEVTRVLMVEKENEI<br>SPPSQGEVPPGPLPEAVLALYNSTRAQVAGESAETEPEPEADYYAKEVTRVLMVEKENEI	61<br>120
H.TGFB1<br>D.TGFB1	YKTVETGSHSIYMFFNTSELRAAVPDPMLLSRAELRLLRLKLSVEQHVELYQKYSNNSW<br>R<br>YKTVETGSHSIYMFFNTSELRAAVPDPMLLSRAELRLLRLKLSVEQHVELYQKYSNNSW<br>R	121<br>180
H.TGFB1<br>D.TGFB1	YLSNRLLTPSDSPEWLSFDVTGVVRQWLSQGGAMEGFRLSAHCSCDSKDNTLRVGINVGF<br>YLSNRLLTPSDSPEWLSFDVTGVVRQWLSQGGAMEGFRLSAHCSCDSKDNTLRVGINVGF	181<br>240
H.TGFB1<br>D.TGFB1	SSSRRGDLATIDGMNRPFLLLMATPLERAQQLHSSRHRRALDTNYCFSSTEKNCCVRQLY<br>SSSRRGDLATIDGMNRPFLLLMATPLERAQQLHSSRHRRALDTNYCFSSTEKNCCVRQLY	241<br>300
H.TGFB1<br>D.TGFB1	IDFRKDLGWKWIHEPKGYHANFCLGPCPYIWSLDTQYSKVLALYNQHNPGASAAPCCVPQ<br>IDFRKDLGWKWIHEPKGYHANFCLGPCPYIWSLDTQYSKVLALYNQHNPGASAAPCCVPQ	301<br>360
H.TGFB1<br>D.TGFB1	VLEPLPIVYYVGRKPKVEQLSNMIVRSCKCS<br>VLEPLPIVYYVGRKPKVEQLSNMIVRSCKCS	361<br>420

Table S2. Comparative amino acid analysis of donkey and horse TGFB2 gene

H.TGFB2<br>D.TGFB2	MHYCVLSAFLLLHLVAVALSLSTCSTLDMDQFMRKRIEAIRGQILSKLKLTSPPEDYPEP<br>MHYCVLSAFLLLHLVAVALSLSTCSTLDMDQFMRKRIEAIRGQILSKLKLTSPPEDYPEP	1<br>60
H.TGFB2<br>D.TGFB2	EEVPPEVISIYNSTRDLLQEKASRRAAACERERSDEEYYAKEVYKIDMPPFFPSENAIPP<br>EEVPPEVISIYNSTRDLLQEKASRRAAACERERSDEEYYAKEVYKIDMPPFFPSENAIPP	61<br>120
H.TGFB2<br>D.TGFB2	TFYRPYFRIVRFDVSAMEKNASNLVKAEFRVFRLQNPKARVPEQRIELYQILKSKDLTSP<br>TFYRPYFRIVRFDVSAMEKNASNLVKAEFRVFRLQNPKARVPEQRIELYQILKSKDLTSP	121<br>180
H.TGFB2<br>D.TGFB2	TQRYIDSKVVKTRAEGEWLSFDVTDAVHEWLHHKDRNLGFKISLHCPCCTFVPSNNYII<br>P<br>TQRYIDSKVVKTRAEGEWLSFDVTDAVHEWLHHKDRNLGFKISLHCPCCTFVPSNNYII<br>P	181<br>240
H.TGFB2<br>D.TGFB2	NKSEELEARFAGIDGTSTYTSGDQKTIKSTRKKNSGKTPHLLLMLLPSYRLESQQSNRRK<br>NKSEELEARFAGIDGTSTYTSGDQKTIKSTRKKNSGKTPHLLLMLLPSYRLESQQSNRRK	241<br>300
H.TGFB2<br>D.TGFB2	KRALDAAYCFRNVQDNCCLRPLYIDFKRDLGWKWIHEPKGYNANFCAGACPYLWSSD<br>TQH<br>KRALDAAYCFRNVQDNCCLRPLYIDFKRDLGWKWIHEPKGYNANFCAGACPYLWSSD<br>TQH	301<br>360
H.TGFB2	SRVLSLYNTINPEASASPCCVSQDLEPLTILYYIGKTPKIEQLSNMIVKSCKCS	361

D.TGFB2	SRVLSLYNTINPEASASPCCVSQDLEPLTILYYIGKTPKIEQLSNMIVKSCKCS	420

Table S3. Comparative amino acid analysis of donkey and horse TGFB3 gene

H.TGFB3<br>D.TGFB3	MKMHLQRALVVLALLNFATVSLSLSTCTTLDFGHIKKKRVEAIRGQILSKLRLTSPPEPS<br>MKMHLQRALVVLALLNFATVSLSLSTCTTLDFGHIKKKRVEAIRGQILSKLRLTSPPEPS	1<br>60
H.TGFB3<br>D.TGFB3	VMTHVPYQVLALYNSTRELLEEMHGEREDGCTQENTESEYYAKEIHKFDMIQGLAEHN<br>EL<br>VMTHVPYQVLALYNSTRELLEEMHGEREDGCTQENTESEYYAKEIHKFDMIQGLAEHN<br>EL	61<br>120
H.TGFB3<br>D.TGFB3	AVCPKGITSKVFRFNVSSVEKNGTNLFRAEFRVLRVPNPSSKRNEQRIELFQILRPDEHI<br>AVCPKGITSKVFRFNVSSVEKNGTNLFRAEFRVLRVPNPSSKRNEQRIELFQILRPDEHI	121<br>180
H.TGFB3<br>D.TGFB3	AKQRYIGGKNLPTRGTAEWLSFDVTDTVREWLLRRESNLGLEISIHCPCHTFQPNGDILE<br>AKQRYIGGKNLPTRGTAEWLSFDVTDTVREWLLRRESNLGLEISIHCPCHTFQPNGDILE	181<br>240
H.TGFB3<br>D.TGFB3	NIHEVMEIKFKGVDSEDDHGRGDLGRLKKQKDHHNPHLILMMIPPHRLDNPGQGGQR<br>KKR<br>NIHEVMEIKFKGVDSEDDHGRGDLGRLKKQKDHHNPHLILMMIPPHRLDNPGQGGQR<br>KKR	241<br>300
H.TGFB3<br>D.TGFB3	ALDTNYCFRNLEENCCVRPLYIDFRQDLGWKWVHEPKGYYANFCSGPCPYLRSADTTHS<br>T<br>ALDTNYCFRNLEENCCVRPLYIDFRQDLGWKWVHEPKGYYANFCSGPCPYLRSADTTHS<br>T	301<br>360
H.TGFB3<br>D.TGFB3	VLGLYNTLNPEASASPCCVPQDLEPLTILYYVGRTPKVEQLSNMVVKSCKCS<br>VLGLYNTLNPEASASPCCVPQDLEPLTILYYVGRTPKVEQLSNMVVKSCKCS	361<br>420

Table S4. Comparative amino acid analysis of donkey and horse TAB1 gene

H.TAB1<br>D.TAB1	MAAQRRSLLQSEQQPSWTDDLPLCHLSGVGSASNRTYSADGKGTESHPPEDSWLKFRSEN<br>MAAQRRSLLQSEQQPSWTDDLPLCHLSGVGSASNRTYSADGKGTESHPPEDSWLKFRSEN	1<br>60
H.TAB1<br>D.TAB1	NCFLYGVFNGYDGTRVTNFVAQRLSAELLLGQLNAEHTEADVRRVLVQAFDVVERSFLES<br>NCFLYGVFNGYDGTRVTNFVAQRLSAELLLGQLNAEHTEADVRRVLVQAFDVVERSFLES	61<br>120
H.TAB1<br>D.TAB1	IDDALAEKASLQSQLPEGVPQHQLPPQYQKILERLKTLEREISGGAMAVVAVLLNNKLYV<br>IDDALAEKASLQSQLPEGVPQHQLPPQYQKILERLKTLEREISGGAMAVVAVLLNNKLYV	121<br>180
H.TAB1<br>D.TAB1	ANVGTNRALLCKSTVDGLQVTQLNVDHTTENEDELFRLSQLGLDAGKIKQVGIICGQEST<br>ANVGTNRALLCKSTVDGLQVTQLNVDHTTENEDELFRLSQLGLDAGKIKQVGIICGQEST	181<br>240
H.TAB1<br>D.TAB1	RRIGDYKVKYGYTDIDLLSSAKSKPIIAEPEIHGAQPLDGVTGFLVLMSEGLYKALEAAH<br>RRIGDYKVKYGYTDIDLLSAAKSKPIIAEPEIHGAQPLDGVTGFLVLMSEGLYKALEAAH	241<br>300
H.TAB1<br>D.TAB1	GPGQANQEIAAMIDTEFAKQTSLDSVAQAVVDRVKRIHGDTFASGGERAKFCPRHEDMTL<br>GPGQANQEIAAMIDTEFAKQTSLDSVAQAVVDRVKRIHGDTFASGGERAKFCPRHEDMTL	301<br>360
H.TAB1<br>D.TAB1	LVRNFGYPLGELSQPTPTPAPAAGGRVYPVSVPYSSAQSTSKTSVTLSLVMPSQGQLVNG<br>LVRNFGYPLGELSQPTPTPAPAAGGRVYPVSVPYSSAQSTSKTSVTLSLVMPSQGQLVNG	361<br>420

H.TAB1<br>D.TAB1	AHSASTLDEATPTLTNQSPTLTLQSTSTHTQSSSSSSDGGLFRSRPAHSLPPGEDGRVEP<br>AHSASTLDEATPTLTNQSPTLTLQSTSTHTQSSSSSSDGGLFRSRPAHSLPPGEDGRVEP	421<br>480
H.TAB1<br>D.TAB1	YVDFAEFYRLWSVDHGEQSVMTAP<br>YVDFAEFYRLWSVDHGEQSVMTAP	481<br>504

Table S5. Comparative amino acid analysis of donkey and horse TAB2 gene

H.TAB2<br>D.TAB2	MAQGSHQIDFQVLHDLRQKFPEVPEVVVSRCMLQNNNNLDACCAVLSQESTRYLYGEGDL<br>MAQGSHQIDFQVLHDLRQKFPEVPEVVVSRCMLQNNNNLDACCAVLSQESTRYLYGEGDL	1<br>60
H.TAB2<br>D.TAB2	NFSDDSGISGLRNHMTSLNLDLQSQNVYHHGREGNRMNGSRTLTHSISDGQLQGGQSNNE<br>NFSDDSGISGLRNHMTSLNLDLQSQNVYHHGREGNRMNGSRTLTHSISDGQLQGGQSNNE	61<br>120
H.TAB2<br>D.TAB2	LFQQEPQTAPAQVPQGFNVFGMSSTSGASNSTPHLGFHLGSKGTSNLSQQTPRFNPIMVT<br>LFQQEPQTAPAQVPQGFNVFGMSSTSGASNSTPHLGFHLGSKGTSNLSQQTPRFNPIMVT	121<br>180
H.TAB2<br>D.TAB2	LAPNIQTGRNTPTSLHIHGVPPPVLNSPQGNSIYIRPYITPPSGTARQTQQHSGWVSQFN<br>LAPNIQTGRNTPTSLHIHGVPPPVLNSPQGNSIYIRPYITPPSGTARQTQQHSGWVSQFN	181<br>240
H.TAB2<br>D.TAB2	PMNPQQVYQPSQPGPWTTYPASNPLSHTSAQQPNQQGHQTSHVYMPISSPTTPQPPTIHS<br>PMNPQQVYQPSQPGPWTTYPASNPLSHTSAQQPNQQGHQTSHVYMPISSPTTPQPPTIHS	241<br>300
H.TAB2<br>D.TAB2	SGSSQSSAHSQYNIQNISTGPRKNQIEIKLEPPQRNSSSKLRSSGPRASSSSPSVNSQTL<br>SGSSQSSAHSQYNIQNISTGPRKNQIEIKLEPPQRNNSSKLRSSGPRTSSSSSSVNSQTL	301<br>360
H.TAB2<br>D.TAB2	NRNQPTVYIAASPPNTDEVMARSQPKVYISANATAGDEQVMRNQPTLFISTNSGASAASR<br>NRNQPTVYIAASPPNTDEVMSRSQPKVYISANATAGDEQVMRNQPTLFISTNSGASAASR	361<br>420
H.TAB2<br>D.TAB2	NMSGQVSMGPAFIHHHPPKSRAVGNNSATSPRVVVTQPNTKYTFKITVSPNKPPAVSPGV<br>NMSGQVSMGPAFIHHHPPKSRAVGNNSATSPRVVVTQPNTKYTFKITVSPNKPPAVSPGV	421<br>480
H.TAB2<br>D.TAB2	VSPTFELANLLNHPDHYVETENIQHLTDPALAHVDRISEARKLSMGSDDAAYTQALLVHQ<br>VSPTFELANLLNHPDHYVETENIQHLTDPALAHVDRISEARKLSMGSDDAAYTQALLVHQ	481<br>540
H.TAB2<br>D.TAB2	KARMERLQRELEIQKKKLDKLKSEVNEMENNLTRRRLKRSNSISQIPSLEEMQQLRSCNR<br>KARMERLQRELEIQKKKLDKLKSEVNEMENNLTRRRLKRSNSISQIPSLEEMQQLRSCNR	541<br>600
H.TAB2<br>D.TAB2	QLQIDIDCLTKEIDLFQARGPHFNPSAIHNFYDNIGFVGPVPPKPKDQRSTIKTPKTQDT<br>QLQIDIDCLTKEIDLFQARGPHFNPSAIHNFYDNIGFVGPVPPKPKDQRSTIKTPKTQDT	601<br>660
H.TAB2<br>D.TAB2	EDDEGAQWNCTACTFLNHPALIRCEQCEMPRHF<br>EDDEGAQWNCTACTFLNHPALIRCEQCEMPRHF	661<br>714

Table S6. Comparative amino acid analysis of donkey and horse TAB3 gene

H.TAB3<br>D.TAB3	MAQSSPQLDMQVLHDLRQRFPEIPEGVVSQCMIQNNNNLEACCRALSQESSKYLYMEYHS<br>MAQSSPQLDMQVLHDLRQRFPEIPEGVVSQCMLQNNNNLEACCRALSQESSKYLYMEYHS	1<br>60
H.TAB3<br>D.TAB3	PDDNRMNRNRLLHINLGIHSPGSYHPGDGAQLNGGRTLVHSSSDGHIDPQHAAGKQLICL<br>PDDNRMNRNRLLHINLGIHSPGSYHPGDGAQLNGGRTLVHSSSDGHIDPQHAAGKQLICL	61<br>120
H.TAB3<br>D.TAB3	VQEPHSAPAVVAATPNYNPFFMNEQNRSAATPPSQPPQQPSSMQTGMNPSAMQGPSPPPP<br>VQEPHSAPAVVAATPNYNPFFMNEQNRSAATPPSQPPQQPSSMQTGMNPSAMQGPSPPPL	121<br>180
H.TAB3<br>D.TAB3	PPPSYMHIPRYSTNPITVTVSQNLPSGQTVPRALQILPQIPSNLYGSPGSIYIRQTSQSS<br>PPPSYMHIPRYSTNPITVTVSQNLPSGQTVPRALQILPQIPSNLYGSPGSIYIRQTSQSS	181<br>240
H.TAB3<br>D.TAB3	PGRQTPQSAPWQSSPQGPVPHYGQRPLPVYPHQQNYQPSQYSPKQQQIPQAAYHSPPPSQ<br>PGRQTPQSAPWQSSPQGPVPHYGQRPLPVYPHQQNYQPSQYSPKQQQIPQAAYHSPPPSQ	241<br>300
H.TAB3<br>D.TAB3	CPSPFSSPQHQVQPSQLGHPSSHVFMPPSPSTTPPHPYQQGPPSYQKQGSHSVAYLPYTT<br>CPSPFSSPQHQVQPSQLGHPSSHVFMPPSPSTTPPHPYQQGPPSYQKQGSHSVAYLPYTT	301<br>360
H.TAB3<br>D.TAB3	SSLPKGSMKKIEITVEPSQRPGTAINRSPSPISNQPSPRNQHSLYTATTPPSSSPSRGIS<br>SSLPKGSMKKIEITVEPSQRPGTAINRSPSPISNQPSPRNQHSLYTATTPPSSSPSRGIS	361<br>420
H.TAB3<br>D.TAB3	SQPKPPFSVNPVYITYTQPTGPSCAPSPSPRVIPNPTTVFKITVGRATTENLLNLVDQEE<br>GQPKPPFSVNPVYITYTQPTGPSCAPSPSPRVIPNPTTVFKITVGRATTENLLNLVDQEE	421<br>480
H.TAB3<br>D.TAB3	RSAAPEPIQPISVIPGSGGEKGSHRYQRSSSSGSDDYAYTQALLLHQRARMERLAKQLKL<br>RSAAPEPIQPISVIPGSGGEKGSHRYQRSSSSGSDDYAYTQALLLHQRARMERLAKQLKL	481<br>540
H.TAB3<br>D.TAB3	EKEELERLKAEVNGMEHDLMQRRLRRVSCSTATPTPEEMTRLRSMNRQLQINVDCTLKEV<br>EKEELERLKAEVNGMEHDLMQRRLRRVSCSTATPTPEEMTRLRSMNRQLQINVDCTLKEV	541<br>600
H.TAB3<br>D.TAB3	DLLQSRGNFDPKAMNNFYDNIEPGPVVPPKPSKKEHLTGSKQSPRTQPRDEDYEGAPWNC<br>DLLQSRGNFDPKAMNNFYDNIEPGPVVPPKPSKKEHLTGSKQSPRTQPRDEDYEGAPWNC	601<br>660
H.TAB3<br>D.TAB3	DSCTFLNHPALNRCEQCEMPRYT<br>DSCTFLNHPALNRCEQCEMPRYT	661<br>714

Table S7. Comparative amino acid analysis of donkey and horse TGIF1 gene

H. TGIF1<br>D. TGIF1	MDIPLDLSSSAGSGKRRRRGNLPKESVQILRDWLYEHRYNAYPSEQEKALLSQQTHLSTL<br>MDIPLDLSSSAGSGKRRRRGNLPKESVQILRDWLYEHRYNAYPSEQEKALLSQQTHLSTL	1<br>60
H. TGIF1<br>D. TGIF1	QVCNWFINARRRLLPDMLRKDGKDPNQFTISRRGAKISEASSVESAMGIKNFMPALEESA<br>QVCNWFINARRRLLPDMLRKDGKDPNQFTISRRGAKISEASSVESAMGIKNFMPALEESA	61<br>120
H. TGIF1<br>D. TGIF1	FHSCTAGPNPALGRPLSPKPSSPGSILPRPSVICHTTVTALKDVPFSLCQPVGVGQNTDI<br>FHSCTAGPNPALGRPLSPKPSSPGSILPRPSVICHTTVTALKDVPFSLCQPVGVGQNTDI	121<br>180
H. TGIF1<br>D. TGIF1	QQIAASSFTDTSLMYPEDTCKSGPSTNTQSGLFNTPPPTPPDLNQDFSGFQLLVDVALKR<br>QQIAASSFTDTSLMYPEDTCKSGPSTNTQSGLFNTPPPTPPDLNQDFSGFQLLVDVALKR	181<br>240

Table S8. Comparative amino acid analysis of donkey and horse TGIF2 gene

H. TGIF2<br>D. TGIF2	MSDSDLGEDEGLLSLAGKRKRRGNLPKESVKILRDWLYLHRYNAYPSEQEKLS LSGQTNL<br>MSDSDLGEDEGLLSLAGKRKRRGNLPKESVKILRDWLYLHRYNAYPSEQEKLS LSGQTNL	1<br>60
H. TGIF2<br>D. TGIF2	SVLQICNWFINARRRLLPDMLRKDGKDPNQFTISRRGGKASDVALPRGSSPSV LAVSVPA<br>SVLQICNWFINARRRLLPDMLRKDGKDPNQFTISRRGGKASDVALPRGSSPSV LAVSVPA	61<br>120
H. TGIF2<br>D. TGIF2	PTNVLSLSVCSVPLHSGQGEKPAAPFPQGELEPSKPLVTPGSTLTLLTRAEAG SPTGGLF<br>PTNVLSLSVCSVPLHSGQGEKPAAPFPQGELEPSKPLVTPGSTLTLLTRAEAG SPTGGLF	121<br>180
H. TGIF2<br>D. TGIF2	NTPPPTPPEQDKEDFSSFQLLVEVALQRAAEMELQKQQDPSPPLLHTPIPLVS ENPK<br>NTPPPTPPEQDKEDFSSFQLLVEVALQRAAEMELQKQQDPSPPLLHTPIPLVS ENPK	181<br>240

Table S9. Comparative amino acid analysis of donkey and horse TGFBR1 gene

H.TGFBR1<br>D.TGFBR1	MEAAAAAPRPRLLLLALAAAAALAPGATALQCFCHLCTKDNFTCVTDGLCFV SVTETTDK<br>MEAAAAAPRPRLLLLALAAAAALAPGATALQCFCHLCTKDNFTCVTDGLCFV SVTETTDK	1<br>60
H.TGFBR1<br>D.TGFBR1	VIHNSMCIAEIDLIPRDRPFVCAPSSKTGSVTTTYCCNQDHCNKIELPTVGP FSGKPSSG<br>61<br>VIHNSMCIAEIDLIPRDRPFVCAPSSKTGSVTTTYCCNQDHCNKIELPTVGP FSGKPSSG	61<br>120
H.TGFBR1<br>D.TGFBR1	LGPVELAAVIAGPVCFVCISLMLMVYICHNRTVIHHRVPNEEDPSLDRPFIS EGTTLKDL<br>LGPVELAAVIAGPVCFVCISLMLMVYICHNRTVIHHRVPNEEDPSLDRPFIS EGTTLKDL	121<br>180
H.TGFBR1<br>D.TGFBR1	IYDMTTSGSGSGLPLLVQRTIARTIVLQESIGKGRFGEVWRGKWRGEEVAVK IFSSREER<br>IYDMTTSGSGSGLPLLVQRTIARTIVLQESIGKGRFGEVWRGKWRGEEVAVK IFSSREER	181<br>240
H.TGFBR1<br>D.TGFBR1	SWFREAEIYQTVMLRHENILGFIAADNKDNGTWTQLWLVSDYHEHGSLFDYL NRYTVTVE<br>SWFREAEIYQTVMLRHENILGFIAADNKDNGTWTQLWLVSDYHEHGSLFDYL NRYTVTVE	241<br>300

H.TGFBR1<br>D.TGFBR1	GMIKLALSTASGLAHLHMEIVGTQGKPAIAHRDLKSKNILVKKNGTCCIADL GLAVRHDS<br>GMIKLALSTASGLAHLHMEIVGTQGKPAIAHRDLKSKNILVKKNGTCCIADL GLAVRHDS	301<br>360
H.TGFBR1<br>D.TGFBR1	ATDTIDIAPNHRVGTKRYMAPEVLDDSINMKHFESFKRADIYAMGLVFWEIA RRCSVGGI<br>ATDTIDIAPNHRVGTKRYMAPEVLDDSINMKHFESFKRADIYAMGLVFWEIA RRCSIGGI	361<br>420
H.TGFBR1<br>D.TGFBR1	HEDYQLPYYDLVPSDPSVEEMRKVVCEQKLRPNIPNRWQSCE<br>HEDYQLPYYDLVPSDPSVEEMRKVVCEQKLRPNIPNRWQSCE	361<br>420

Table S10. Comparative amino acid analysis of donkey and horse TGFBR2 gene

H.TGFBR2<br>D.TGFBR2	MGRGLLRGLWPLHIVLWTRIASTIPPQVQKSVNNDIMVTDNDGAVKFPQFCK<br>FCNVSSSI<br>MGRGLLRGLWPLHIVLWTRIASTIPPQVQKSVNNDMMVTDNDGAVKFPQFCK<br>FCNVSSSI	1<br>60
H.TGFBR2<br>D.TGFBR2	CDNQKSCMSNCSITSRCEKPHEVCVAVWRKNDKNITLETVCHDPKLTYHGFV<br>LEDAASPK<br>CDNQKSCMSNCSITSRCEKPREVCVAVWRKNDKNITLETVCHDPKLTYHGFV<br>LEDAASPK	61<br>120
H.TGFBR2<br>D.TGFBR2	CIMKEKKVSGETFFMCSCSSDECNDHIIFSEVNNDIMITDNNGAVKLPQLCK<br>FCDVTSST<br>CIMKEKKVSGETFFMCSCSSDECNDHIIFSEVNNDIMITDNNGAVKLPQLCK<br>FCDVTSST	121<br>180
H.TGFBR2<br>D.TGFBR2	CDNQKSCMSNCSITSICEDPHEVCVAVWRKNDKNITLETVCHDPKLTYHGFV<br>LEDAASPK<br>CDNQKSCMSNCSITSICEDPHEVCVAVWRKNDKNITLETVCHDPKLTYHGFV<br>LEDAASPK	181<br>240
H.TGFBR2<br>D.TGFBR2	CIMKEKKVSGETFFMCSCSSDECNDHIIFSEEYATNNPDLLLVIFQVTGVSL<br>LPPLGIAI<br>CIMKEKKVSGETFFMCSCSSDECNDHIIFSEEYATNNPDLLLVIFQVTGVSL<br>LPPLGIAI	241<br>300
H.TGFBR2<br>D.TGFBR2	AVIITFYCYRVHRQQKLSPSWETSKPRKLMEFSEHLAIILEDDRSDISSTCA<br>NNINHNTE<br>AVIITFYCYRVHRQQKLSPSWETSKPRKLMEFSEHLAIILEDDRSDISSTCA	301<br>360

	NNINHNTE	
H.TGFBR2<br>D.TGFBR2	LLPIELDTLVGKGRFAEVYKAKLKQNTSEQFETVAVKIFPYEEYASWKTEKD<br>IFSDINLK<br>LLPIELDTLVGKGRFAEVYKAKLKQNTSEQFETVAVKIFPYEEYASWKTEKD<br>IFSDINLK	361<br>420
H.TGFBR2<br>D.TGFBR2	HENILQFLTAEERKTELGKQYWLITAFHAKGNLQEYLTRHVISWEDLRKLGS<br>SLARGIAH<br>HENILQFLTAEERKTELGKQYWLITAFHAKGNLQEYLTRHVISWEDLRKLGS<br>SLARGIAH	421<br>480
H.TGFBR2<br>D.TGFBR2	LHSDHTLCGRPKMPIVHRDLKSSNILVKNDLTCCLCDFGLSLRLDPTLSVDD<br>LANSGQVG<br>LHSDHTLCGRPKMPIVHRDLKSSNILVKNDLTCCLCDFGLSLRLDPTLSVDD<br>LANSGQVG	481<br>540
H.TGFBR2<br>D.TGFBR2	TARYMAPEVLESRMNLENVESFKQTDVYSMALVLWEMTSRCNAVGEVKDYEP<br>PFGSKVRE<br>TARYMAPEVLESRMNLENVESFKQTDVYSMALVLWEMTSRCNAVGEVKDYEP<br>PFGSKVRE	541<br>620
H.TGFBR2<br>D.TGFBR2	HPCVESMKDNVLRDRGRPEIPSSWLNHQGIQMVCETLTECWDHDPEARLTAQ<br>CVAERFSE<br>HPCVESMKDNVLRDRGRPEIPSSWLNHQGIQMVCETLTECWDHDPEARLTAQ<br>CVAERFSE	621<br>680
H.TGFBR2<br>D.TGFBR2	LEHMDRLSGRSCSEEKIPEDGSLNTTK<br>LEHMDRLSGRSCSEEKIPEDGSLNTTK	681<br>740

Table S11**.** Comparative amino acid analysis of donkey and horse TGFBR3 gene

H. TGFBR3<br>D. TGFBR3	MASHYVVAMFALMSSCLATAGPEPSTQCELSPVNASHPVQALMESFTVLSGCASRGTTGL<br>MASHYVVAMFALMSSCLATAGPEPSTQCELSPVNASHPVQALMESFTVLSGCASRGTTGL	1<br>60
H. TGFBR3<br>D. TGFBR3	PQEVHVLNLRAADPGPGQPQREVTLHLNPISSVHIHHKPVVFLLNSPQPLVWHLKTERLA<br>PQEVHVLNLRAADPGPGQPQREVTLHLNPISSVHIHHKPVVFLLNSPQPLVWHLKTERLA	61<br>120
H. TGFBR3<br>D. TGFBR3	IGVSRLFLVSEGSVVHFSSGNFSLSAETEERSFPHGNEHLLNWARKEYGAVTSFTELKIA<br>IGVSRLFLVSEGSVVHFSSGNFSLSAETEERSFPHGNEHLLNWARKEYGAVTSFTELKIA	121<br>180
H. TGFBR3<br>D. TGFBR3	RNIYIKVGEDQVFPPTCNIGKNFLSLNYLAEYLQPKAAEGCVISSQPQDKEVHIIELITP<br>RNIYIKVGEDQVFPPTCNIGKNFLSLNYLAEYLQPKAAEGCVISSQPQDKEVHIIELITP	181<br>240
H. TGFBR3<br>D. TGFBR3	NSNPYSAFQVDIVIDIRPSRKDPEVVKNLILILKCKKSVNWVIKSFDVKGNLKVIAPNSI<br>NSNPYSAFQVDIVIDIRPSRKDPEVVKNLILILKCKKSVNWVIKSFDVKGNLKVIAPNSI	241<br>300
H. TGFBR3<br>D. TGFBR3	GFGKESERSMIMTKSIRDDIPSTQENLVKWALDNGYSPVTSYTVAPVANRFHLRLENNEE<br>GFGKESERSMIMTKSIRDDIPSTQENLVKWALDNGYSPVTSYTVAPVANRFHLRLENNEE	301<br>360
H. TGFBR3<br>D. TGFBR3	MRDEEIHTIPPELRILLDPGILPALDNPPIRGGGGRNGGFPFPFPDISRRGQKEGGEDGI<br>MRDEEIHTIPPELRILLDPGILPALDNPPIRGGGGRNGGFPFPFPDISRRGQKEGGEDGI	361<br>420
H. TGFBR3<br>D. TGFBR3	PRPKDPVIPSIRLFPGPREPEEVQGSMDVALSVKCDNEKMTVAVEKDSFQANSYSGMELT<br>PRPKDPVIPSIRLFPGPREPEEVQGSMDVALSVKCDNEKMTVAVEKDSFQANSYSGMELT	421<br>480
H. TGFBR3<br>D. TGFBR3	LLDPTCKAKMNGTHFILESPLNGCGTRHRRSAPDGVVYYNSIVIQVPSLGDSSGWPDGYE<br>LLDPTCKAKMNGTHFILESPLNGCGTRHRRSAPDGVVYYNSIVIQVPSLGDSSGWPDGYE	481<br>540
H. TGFBR3<br>D. TGFBR3	DLESGDNGFPGDTDEGETSFFSRPEIVVFNCSLRQVGKPSSFQDPPNRNITFNMELYNTD<br>DLESGDNGFPGDTDEGETSFFSRPEIVVFNCSLRQVGKPSSFQDPPNRNITFNMELYNTD	541<br>600
H. TGFBR3<br>D. TGFBR3	LFLVPSQGIFSVAENGHIYVEVSVTKADQELGFAIQTCFISPYSNSEGMSDYTIIENICP<br>LFLVPSQGIFSVAENGHIYVEVSVTKADQELGFAIQTCFISPYSNSEGMSDYTIIENICP	601<br>660
H. TGFBR3<br>D. TGFBR3	KDESVKFYNPKRVHFPIPQAEMDKKRFSFVFKSVFNSSLLFLQCELTLCTKKEKDPQKLP<br>KDESVKFYNPKRVHFPIPQAEMDKKRFSFVFKSVFNSSLLFLQCELTLCTKKEKDPQKLP	661<br>714

H. TGFBR3<br>D. TGFBR3	KCVPPDEACTSLDASMIWAMMQNKKTFTKPLAVIHHQVESKETGPSIKEPSPISPPIFHG<br>KCVPPDEACTSLDASMIWAMMQNKKTFTKPLAVIHHQVESKETGPSIKEPSPISPPIFHG	601<br>660
H. TGFBR3<br>D. TGFBR3	LDTLTVMGIAFAAFVIGALLTGALWYIYSHTGETAGRQQVPTSPPASENSSAAHSIGSTQ<br>LDTLTVMGIAFAAFVIGALLTGALWYIYSHTGETAGRQQVPTSPPASENSSAAHSIGSTQ	661<br>714

H. TGFBR3<br>D. TGFBR3	STPCSSSSTA<br>STPCSSSSTA	661<br>714

Table S12. Comparative amino acid analysis of donkey and horse GDF1 gene

H.GDF1<br>D.GDF1	MPPPRRGPGRRVLFLLLALLLPSPPPARAPAPPGPAAALLQALGLRDAPRDAPTPRPVPP<br>MPPPRRGPGRRVLFLLLALLLPSPPPARAPAPPGPAAALLQALGLRDAPRDAPTPRPVPP	1<br>60
H.GDF1<br>D.GDF1	VMWHLFRRRDPQESRTNLRRTPPGATLRPCHLEELGVAGNIVRHVLDRGAPARPPEPASA<br>VMWHLFRRRDPQESRTNLRRTPPGATLRPCHLEELGVAGNIVRHVLDRGAPARPPEPASA	61<br>120
H.GDF1<br>D.GDF1	AGHCPEWTVVFDLSAVEPAERPSRARLELRFAAAEATAGGWELSVARAAEGAGPGPVLLR<br>AGHCPEWTVVFDLSAVEPAERPSRARLELRFAAAEATAGGWELSVARAAEGAGPGPVLLR	121<br>180
H.GDF1<br>D.GDF1	QAVLALGTPVRAELLGAAWARNASAPRSLRLSLALRPRVPAACARLAEASLLLATLDPRL<br>QAVPALGTPVRAELLGAAWARNASAPRSLRLSLALRPRVPAACARLAEASLLLATLDPRL	181<br>240
H.GDF1<br>D.GDF1	CHPLARPRREAEPAVGGGAGGACRARRLYVSFREVGWHRWVIAPRGFLANYCQGQCALPA<br>CHPLARPRREAEPAVGGGPGGACRARRLYVSFREVGWHRWVIAPRGFLANYCQGQCALPA	241<br>300
H.GDF1<br>D.GDF1	ALSGPGGPPALNHAVLRALMHAAAPGAAGLPCCVPARLSPISVLFFDNSDNVVLRHYEDM<br>ALSGPGGPPALNHAVLRALMHAAAPGAAGLPCCVPARLSPISVLFFDNSDNVVLRHYEDM	301<br>360
H.GDF1<br>D.GDF1	VVDECGCR<br>VVDECGCR	361<br>420

Table S13. Comparative amino acid analysis of donkey and horse GDF2 gene

H.GDF2<br>D.GDF2	MCRGALRVALLALLACSAQGKPLESRGRAAGGGDAHRPRGGPGGEQEAGTFDLRMFLENM<br>MCRGALRVALLALLACSAQGKPLESRGRAAGGGDAHRPRGGPGGEQEAGTFDLRMFLENM	1<br>60
H.GDF2<br>D.GDF2	KVDFLRSLNLSGVPSQDKTRAEPPQYMIDLYNRYTTDKSSTPTSNIVRSFSVEDAVSVMA<br>KVDFLRSLNLSGVPSQDKTRAEPPQYMIDLYNRYTTDKSSTPTSNIVRSFSVEDAVSVMA	61<br>120
H.GDF2<br>D.GDF2	TEDLSFQKHILFFNVSIPRHEQITRAELRLHISCQSHVDSSHELKGNMVIYDVLDGADAW<br>TEDLSFQKHILFFNISIPRHEQITRAELRLHISCQSHVDSSHELKGNMVIYDVLDGADAW	121<br>180
H.GDF2<br>D.GDF2	DTSMGTKTFLVSQDIRDEGWETFEVSSAVKRWVRADSTKSKNKLEVTVESHRKGCDRLDI<br>DASMGTKTFLVSQDIRDEGWETFEVSSAVKRWVRADSTKSKNKLEVTVESHRKGCDRLDI	181<br>240
H.GDF2<br>D.GDF2	SVPPGSKNLPFFVVFSNDRSNGTKETRLELREMIGHEQESVLRKLSKDGLAEADENKDEE<br>SVPPGSKNLPFFVVFSNDRSNGTKETRLELREMIGHEQESVLRKLSKDGLAEADENKDEE	241<br>300
H.GDF2<br>D.GDF2	DVEGSMAAGSSLARRKRSAGAGNHCQKTSLRVNFEDIGWDSWIIAPKEYDAYECKGGCFF<br>DVEGSMAVGSSLARRKRSAGAGNHCQKTSLRVNFEDIGWDSWIIAPKEYDAYECKGGCFF	301<br>360
H.GDF2<br>D.GDF2	PLADDVTPTKHAIVQTLVHLKFPMKVGKACCVPTKLSPISILYKDDMGVPTLKYHYEGMS<br>PLADDVTPTKHAIVQTLVHLKFPMKVGKACCVPTKLSPISILYKDDMGVPTLKYHYEGMS	361<br>420
H.GDF2<br>D.GDF2	VAECGCR<br>VAECGCR	421<br>480

Table S14. Comparative amino acid analysis of donkey and horse GDF3 gene

H. GDF3<br>D. GDF3	MIPSLPALALGLLLTLALGQTFQFQENVFLQFLGLDKVPSPQKFQPVPYILKKIFQDREA<br>MIPSLPVLALGLMLTLALGQTFQFQENVFLQFLGLDKVPSPQKFQPVPYILKKIFQDREA	1<br>60
H. GDF3<br>D. GDF3	AATTGGSQDLCSIKNLGVRGNVLRLLPDQGFYLYSKDLPQASCLQKLLYFNLAAIRDKEQ<br>AATTGGSQDLCSIKNLGVRGNVLRLLPDQGFYLYSKDLPQASCLQKLLYFNLAAIRDKEQ	61<br>120
H. GDF3<br>D. GDF3	LTMAQLGLDLGSNTYYNLGPELELALSLVQEPRVWGQSISTPGKPFALQSVPWPQGVLRF<br>LTMAQLGLDLGSNTYYNLGPELELALSLVQEPRVWGQSISTPGKPFALQSVPWPQGVLHF	121<br>180
H. GDF3<br>D. GDF3	NLLDVAKDWNNNPQKNLGLFLEILVKGDRDFGVNFQLEDTCARLRHSLHASLLVVTLNPE<br>NLLDVAKDWNNNPQKNLGLLLEILVKGDRDFGVNFQLEDTCARLRHSLHASLLVVTLNPE	181<br>240
H. GDF3<br>D. GDF3	QCHPSSRKRRSAIPAPNASCKNLCHRHQLFINFRDLGWHKWIIAPKGFMANYCHGDCPFS<br>QCHPSSRKRRSAIPAPNASCKNLCHRHQLFINFRDLGWHKWIIAPKGFMANYCHGDCPFS	241<br>300
H. GDF3<br>D. GDF3	LTTSLNSSNYAFMQALMHAVDPQIPQAVCIPTKLSPISMLYQDNDDNVILRHYEDMVVDE<br>LTTSLNSSNYAFMQALMHAVDPQIPQAVCIPTKLSPISMLYQDNDDNVILRHYEDMVVDE	301<br>360
H. GDF3<br>D. GDF3	CGCG<br>CGCG	361<br>420

Table S15. Comparative amino acid analysis of donkey and horse GDF5 gene

H. GDF5		1
D. GDF5	MRLPKLLTFLLWHLAWLDLEFICTVLGAPDLGQRPQGARPGLAKAEAKERPPLARNIFRP	60
	MRLPKLLTFLLWHLAWLDLEFICTVLGAPDLGQRPQGARPGLAKAEAKERPPLARNIFRP	
H. GDF5	GGHSYGGGATSARAKGGTGQTGGPTQPKKDEPKKLPPRPGGSEPKPGHPPQTRQAATRTV	61
D. GDF5	GGHSYGGGATSARAKGGTGQTGGPTQPKKDEPKKLPPRPGGSEPKPGHPPQTRQAATRTV	120
H. GDF5	TPKGQLPGGKAPPKAGSVPSPFLLKKAREPGPPREPKEPFRPPPITPHEYMLSLYRTLSD	121
D. GDF5	TPKGQLPGGKAPPKAGSVPSPFLLKKAREPGPPREPKEPFRPPPITPHEYMLSLYRTLSD	180
H. GDF5	ADRKGGNSSVKLEAGLANTITSFIDKGQDDRGPVVRKQRYVFDISALEKDGLLGAELRIL	181
D. GDF5	ADRKGGNSSVKLEAGLANTITSFIDKGQDDRGPVVRKQRYVFDISALEKDGLLGAELRIL	240
H. GDF5	RKKSSDTAKPGAPSSRRAAQLKLSSCPSGRQPAALLDVRSVPGLDGSGWEVFDIWKLFRN	241
D. GDF5	RKKSSDTAKPGAPSSRRAAQLKLSSCPSGRQPAALLDVRSVPGLDGSGWEVFDIWKLFRN	300
H. GDF5	FKNSAQLCLELEAWERGRAVDLRGLGFDRTARQVHEKALFLVFGRTKKRDLFFNEIKARS	301
D. GDF5	FKNSAQLCLELEAWERGRAVDLRGLGFDRTARQVHEKALFLVFGRTKKRDLFFNEIKARS	360
H. GDF5	GQDDKTVYEYLFSQRRKRRAPLATRQGKRPTKNPKARCSRKALHVNFKDMGWDDWIIAPL	361
D. GDF5	GQDDKTVYEYLFSQRRKRRAPLATRQGKRPTKNPKARCSRKALHVNFKDMGWDDWIIAPL	420
H. GDF5	EYEAFHCEGLCEFPLRSHLEPTNHAVIQTLMNSMDPESTPPTCCVPTRLSPISILFIDSA	421
D. GDF5	EYEAFHCEGLCEFPLRSHLEPTNHAVIQTLMNSMDPESTPPTCCVPTRLSPISILFIDSA	480
H. GDF5	NNVVYKQYEDMVVESCGCR	481
D. GDF5	NNVVYKQYEDMVVESCGCR	540

Table S16. Comparative amino acid analysis of donkey and horse GDF6 gene

H. GDF6<br>D. GDF6	MDTPRVLLSAVFLISFLWDLPGFQQASISSSSSAELGSAKGIRSRREGKMPRAPQESATA<br>MDTPRVLLSAVFLISFLWDLPGFQQASISSSSSAELGSAKGIRSRREGKMPRAPQESATA	1<br>60
H. GDF6<br>D. GDF6	QAPLERQEHQPQRQDEPRRRRPPKQHQAQESPGRGPRVVPHEYMLSIYRTYSIAEKLGIN<br>QAPLERQEHQPQPQDEPRRRRPPKQHQAQESPGRGPRVVPHEYMLSIYRTYSIAEKLGIN	61<br>120
H. GDF6<br>D. GDF6	ASFFQSSKSANTITSFVDRGLDDLSHTPLRRQKYLFDVSTLSDKEELVGAELRLFRQAPA<br>ASFFQSSKSANTITSFVDRGLDDLSHTPLRRQKYLFDVSTLSDKEELVGAELRLFRQAPA	121<br>180
H. GDF6<br>D. GDF6	VPWGPPAGPLHVQLFPCLSPLQLDARTLDPQGAPRAGWEVFDVWQGLRHQPWKQLCLELR<br>VPWGPPAGPLHVQLFPCLSPLQLDARTLDPQGAPRAGWEVFDVWQGLRHQPWKQLCLELR	181<br>240
H. GDF6<br>D. GDF6	ASWGELGAGEDEARAPGPQQPPPPDLRSLGFGRRVRPPQERALLVVFTRSQRKNLFAEMR<br>ASWGELGAGEDEARAPGPQQPPPPDLRSLGFGRRVRPPQERALLVVFTRSQRKNLFAEMR	241<br>300
H. GDF6<br>D. GDF6	EQLGSAEVAGPSGGAEGSWPPPSGSPDVGPWLPSPGRRRRRTAFASRHGKRHGKKSRLRC<br>EQPGSAEVAGPSGGAEGSWPPPSGSPDAGPWLPSPGRRRRRTAFASRHGKRHGKKSRLRC	301<br>360
H. GDF6<br>D. GDF6	SKKPLHVNFKELGWDDWIIAPLEYEAYHCEGVCDFPLRSHLEPTNHAIIQTLMNSMDPGS<br>SKKPLHVNFKELGWDDWIIAPLEYEAYHCEGVCDFPLRSHLEPTNHAIIQTLMNSMDPGS	361<br>420
H. GDF6<br>D. GDF6	TPPSCCVPTKLTPISILYIDAGNNVVYKQYEDMVVESCGCR<br>TPPSCCVPTKLTPISILYIDAGNNVVYKQYEDMVVESCGCR	421<br>480

Table S17. Comparative amino acid analysis of donkey and horse GDF7 gene

H. GDF7<br>D. GDF7	MDLSAAAALCLWLLSACRPRDGLEAAAVLRAAGAGPAGSPGGGGGGGGRTLAAAAGASTG<br>MDLSAAAALCLWLLSACRPRDGLEAAAVLRAAGAGPAGSPGGGGGGGGRTLAAAAGASTG	1<br>60
H. GDF7<br>D. GDF7	PAAAAPGARAARRAASSGFRNGSVVPHQFMMSLYRSLAGRAPAGAAAASTSGSGRHGRAD<br>PAAAAPGARAARRAASSGFRNGSVVPHQFMMSLYRSLAGRAPAGAAAASTSGSGRHGRAD	61<br>120
H. GDF7<br>D. GDF7	TITGFADQATQDESAAETGQSFLFDVSSLSDADEVVGAELRVLRREFPEPSSGSAIPPPL<br>TITGFADQATQDESAAETGQSFLFDVSSLSDADEVVGAELRVLRRDSPEPSSGSAIPPPL	121<br>180
H. GDF7<br>D. GDF7	LLLLSTCPGAASAPLLLHSRAAEPLDGARWEVFDVADAVRRHRREPRATRVFCLLLRAAA<br>LLLLSTCPGAASAPLLLHSRAAEPLDGARWEVFDVADAVRRHRREPRATRMFCLLLRAAA	181<br>240
H. GDF7<br>D. GDF7	GPARGPLALQLLGFGSRGGGGAAAEERALLVVSSRTQRKESLFREIRAQARALGAPLAAE<br>GPARGPLALQLLGFGSRGGGGAAAEERALLVVSSRTQRKESLFREIRAQARALGAALAAE	241<br>300
H. GDF7<br>D. GDF7	PPPDPGPGTGSPTAVIGGRRRRRTALAGTRAAQGSGGGAGRGHGRRGRSRCSRKPLHVDF<br>PPPDPGPGTGSPTAVIGGRRRRRTALAGTRAAQGSGGGAGRGHGRRGRSRCSRKPLHVDF	301<br>360
H. GDF7<br>D. GDF7	KELGWDDWIIAPLDYEAYHCEGVCDFPLRSHLEPTNHAIIQTLLNSMAPDAAPASCCVPA<br>KELGWDDWIIAPLDYEAYHCEGVCDFPLRSHLEPTNHAIIQTLLNSMAPDAAPASCCVPA	361<br>420
H. GDF7<br>D. GDF7	RLSPISILYIDAANNVVYKQYEDMVVEACGCR<br>RLSPISILYIDAANNVVYKQYEDMVVEACGCR	421<br>480

Table S18. Comparative amino acid analysis of donkey and horse GDF9 gene

H. GDF9<br>D. GDF9	MALPSKFFLWFCCSAWLCFPISLGSQASREAAQIAASAELESEAEPWSLLQPLNGGNRSG<br>MALPSKFFLWFCCSAWLCFPISLGSQASREAAQIAASAELESEAEPWSLLQPLNGGNRSG	1<br>60
H. GDF9<br>D. GDF9	LLPALFKVLYDGQGGAPRLQPDSRALRYMKRLYKAYATKEGIPKSNRGHLYNTVRLFTPC<br>LLPALFKVLYDGQGGAPRLQPDSRALGYMKRLYKAYATKEGIPKSNRGHLYNTVRLFTPY	61<br>120
H. GDF9<br>D. GDF9	AQHKQAPGDQVAGTLPSVDLLFNLDCVTAVEHLLKSVLLYTFNNSVSFPSAVKCVCNLVI<br>AQHKQAPGDQVAGTLPSVDLLFNLDCVTAVEHLLKSVLLYTFNNSVSFPSAVKCVCNLVI	121<br>180
H. GDF9<br>D. GDF9	KEPESKTLPGTPYSFTFNSQFEFRKKYKWIEMDVTPLLQPLVASNKKSIHMSVNLTCGKD<br>KEPESKTLPGTPYSFTFNSQFEFRKKYKWIEMDVTPLLQPLVASNKKSIHMSVNLTCGKD	181<br>240
H. GDF9<br>D. GDF9	QLQHPSAQDSPLNTTLLLFPSLLLYLNDTSAQAYHRWHSLHYKRRPSQGPDQKRDLSACP<br>QLQHPSAQDSPLNTTLLLFPSLLLYLNDTSAQAYHRWHSLHYKRRPSQGPDQKRDLSACP	241<br>300
H. GDF9<br>D. GDF9	EGEGAAEGITSSRHRRSQEAVSSELKKPLVPASLNLSEYFKQFLFPQNECELHDFRLSFS<br>EGEGAAEGIRSSRHRRSQEAVSSELKKPLVPASLNLSEYFKQFLFPQNECELHDFRLSFS	301<br>360
H. GDF9<br>D. GDF9	QLKWDNWIVAPQRYNPRYCKGDCPRAVGHRYGSPVHTMVQNIIHEKLDSSVPRPSCVPAK<br>QLKWDNWIVAPQRYNPRYCKGDCPRAVGHRYGSPVHTMVQNIIHEKLDSSVPRPSCVPAK	361<br>420
H. GDF9<br>D. GDF9	YSPLSVLTIESDGSITYKEYEDMIATKCTCR<br>YSPLSVLTIESDGSITYKEYEDMIATKCTCR	421<br>480

Table S19. Comparative amino acid analysis of donkey and horse GDF10 gene

H.GDF10<br>D.GDF10	MARGPARTSPGPGPQLLPLLPLLLLLLRDAGGSHTVPARSALPAAADGLVGHKDAQWPLG<br>MARGPARTSPGPGPQLLPLLPLLLLLLQDAGGSHTVPARSALPAAADGLVGHKDAQWPLG	1<br>60
H.GDF10<br>D.GDF10	DAAAALGPGARDMVAVHMLRLYEKYSRRGARPGGGNTVRSFRARLEVVNQKAVYFFNLTS<br>DEAAALGPGARDMVAVHMLRLYEKYSRRGARPGGGNTVRSFRARLEVVNQKAVYFFNLTS	61<br>120
H.GDF10<br>D.GDF10	MQDSEMILTATFHFYSEPRWPQAREVPCKQRAKNASCRLLPPGPPARQHLLFRSLSQNTA<br>MQDSEMILTATFHFYSEPRWPQAREVPCKQRAKNASCRLLPPGPPARQHLLFRSLSQNTA	121<br>180
H.GDF10<br>D.GDF10	TQGLLRGAMALAPPPRGLWQAKDISPIVKAARRDGELLLSAQLDAGEKDPGVPRPSPHAP<br>TQGLLRGAMALAPPPRGLWQAKDISPIVKAARRDGELLLSAQLDPGEKDPGVPRPSPHAP	181<br>240
H.GDF10<br>D.GDF10	YILVYANDLAISEPNSVAVTLQRYDPFQTGDPEPGAAPNSSADPRVRRATQVTGPLQDNE<br>YILVYANDLAISEPNSVAVTLQRYDPFQTGDPEPGAAPNSSADPRVRRATQVTGPLQDNE	241<br>300
H.GDF10<br>D.GDF10	LPGLDERPAHGPHAQPYHKHELWPSPFRALKSRPGRKDRRRKGQDAFAASSQVLDFDEKT<br>LPGLDERPAHGPHAQPYHKHELWPSPFRALKSRPGRKDRRRKGQDALPASSQVLDFDEKT	301<br>360
H.GDF10<br>D.GDF10	MQKARRKQWDEPRVCSRRYLKVDFADIGWNEWIISPKSFDAYYCAGACEFPMPKIVRPSN<br>MQKARRKQWDEPRVCSRRYLKVDFADIGWNEWIISPKSFDAYYCAGACEFPMPKIVRPSN	361<br>420
H.GDF10<br>D.GDF10	HATIQSIVRAVGIVPGIPEPCCVPDKMNSLGVLFLDENRNVVLKVYPNMSVETCACR<br>HATIQSIVRAVGIVPGIPEPCCVPDKMNSLGVLFLDENRNVVLKVYPNMSVETCACR	421<br>480

Table S20. Comparative amino acid analysis of donkey and horse GDF11 gene

H.GDF11<br>D.GDF11	MVLAAPLLLGFLLLALELRPRGEAAEGPAAAAAAAAAA-GAGGERSSRPAPSVAPEPDGC<br>MVLAAPLLLGFLLLALELRPRGEAAEGPAAAAAAAAAAAGAGGERSSRPAPSVAPEPDGC	1<br>60
H.GDF11<br>D.GDF11	PVCVWRQHSRELRLESIKSQILSKLRLKEAPNISREVVKQLLPKAPPLQQILDLHDFQGD<br>PVCVWRQHSRELRLESIKSQILSKLRLKEAPNISREVVKQLLPKAPPLQQILDLHDFQGD	61<br>120
H.GDF11<br>D.GDF11	ALQPEDFLEEDEYHATTETVISMAQETDPAVQTDGSPLCCHFHFSPKVMFTKVLKAQLWV<br>ALQPEDFLEEDEYHATTETVISMAQETDPAVQTDGSPLCCHFHFSPKVMFTKVLKAQLWV	121<br>180
H.GDF11<br>D.GDF11	YLRPVPRPATVYLQILRLKPLTGEGTAGGGGGGRRHIRIRSLKIELHSRSGHWQSIDFKQ<br>YLRPVPRPATVYLQILRLKPLTGEGTAGGGGGGRRHIRIRSLKIELHSRSGHWQSIDFKQ	181<br>240
H.GDF11<br>D.GDF11	VLHSWFRQPQSNWGIEINAFDPSGTDLAVTSLGPGAEGLHPFMELRVLENTKRSRRNLGL<br>VLHSWFRQPQSNWGIEINAFDPSGTDLAVTSLGPGAEGLHPFMELRVLENTKRSRRNLGL	241<br>300
H.GDF11<br>D.GDF11	DCDEHSSESRCCRYPLTVDFEAFGWDWIIAPKRYKANYCSGQCEYMFMQKYPHTHLVQQA<br>DCDEHSSESRCCRYPLTVDFEAFGWDWIIAPKRYKANYCSGQCEYMFMQKYPHTHLVQQA	301<br>360
H.GDF11<br>D.GDF11	NPRGSAGPCCTPTKMSPINMLYFNDKQQIIYGKIPGMVVDRCGCS<br>NPRGSAGPCCTPTKMSPINMLYFNDKQQIIYGKIPGMVVDRCGCS	361<br>420

Table S21. Comparative amino acid analysis of donkey and horse GDF15 gene

H.GDF15	MRHFPMLLLSWLLLWLPPGGALPLTQDHSPAFLGPSGGHSSLDVSRFRELRERYEHLQAR	1
D.GDF15	MRHFPMLLLSWLLLWLPPGGALPLTQDHSPAFLGPSGGHSSLDVSRFRELRKRYEHLQAR	60
H.GDF15	LLLNQTQEDWNADPIPVDHVRMLTPKLRLGPDGHLRLHALRADLTEGLPAGSRLRQALLR	61
D.GDF15	LLLNQTQEDWNADPIPVDHVRILTPKLRLGPDGHLRLHALRADLTEGLPAGSRLRQALLR	120
H.GDF15	LSPQAPGSWDLTRQLQRQLRLGGPAAPALSLRLPRRGGPSPEALRAAQPRLELRWWPPAA	121
D.GDF15	LSPQAPGSWDLTRQLQRQLRLGGPAAPALSLRLPRLGGPSPEARRAAQPRLELRWRPPAA	180
H.GDF15	RGRRGAHAHARDACPLGEGRCCRLLSLRASIEDLGWADWVVAPRELDVRMCVGACPGRFR	181
D.GDF15	RGRRGAHAHARDECPLGEGRCCRLLSLRASIEDLGWADWVVAPRELDVRMCVGACPGRFR	240
H.GDF15	SASRHAEAQARLHGLKPAAAPAPCCVPAGYAPVVLLHRAADGRVALTPFDDLVAAGCHCQ	241
D.GDF15	SASRHAEAQARLHGLKPAAAPAPCCVPAGYAPVVLLHRAADGRVALTPFDDLVAAGCHCQ	300

Table S22. Comparative amino acid analysis of donkey and horse BMP1 gene

H. BMP1<br>D. BMP1	MPGVARLPLPLPLLLWLLLLARPGRPLDLADYTYELGEEDDSEPLNYKDPCKAVAFLGDI<br>MPGVARLPLPLPLLLWLLLLARPGRPLDLADYTYELGEEDDSEPLNYKDPCKAAAFLGDI	1<br>60
H. BMP1<br>D. BMP1	ALDEEDLRAFQVQQAVDLGQQAIRRSSIKAAENSSTPICQSTSGQPQRKNRGRWRSRFRS<br>ALDEEDLRAFQVQQAVDLGQQAIRRSSIKAAENSSTPICQSTSGQPQRKNRGRWRSRSRS	61<br>120
H. BMP1<br>D. BMP1	RRAATSRPERVWPDGVIPFVIGGNFTGSQRAVFRQAMRHWEKHTCVTFLERTDEDSYIVF<br>RRAATSRPERVWPDGVIPFVIGGNFTGSQRAVFRQAMRHWEKHTCVTFLERTDEDSYIVF	121<br>180
H. BMP1<br>D. BMP1	TYRPCGCCSYVGRRGGGPQAISIGKNCDKFGIVVHELGHVIGFWHEHTRPDRDRHVSIVR<br>TYRPCGCCSYVGRRGGGPQAISIGKNCDKFGIVVHELGHVIGFWHEHTRPDRDRHVSIVR	181<br>240
H. BMP1<br>D. BMP1	ENIQPGQEYNFLKMELQEVESLGENYDFDSIMHYARNTFSRGIFLDTIVPKYEVNGVKPP<br>ENIQPGQEYNFLKMELQEVESLGENYDFDSIMHYARNTFSRGIFLDTIVPKYEVNGVKPP	241<br>300
H. BMP1<br>D. BMP1	IGQRTRLSKGDIAQARKLYKCPACGETLQDSTGNFSSPEYPNGYSAHMHCVWRISVTPGE<br>IGQRTRLSKGDIAQARKLYKCPACGETLQDSTGNFSSPEYPNGYSAHMHCVWRISVTPGE	301<br>360
H. BMP1<br>D. BMP1	KIILNFTSMDLYRSRLCWYDYVEVRDGFWRKAPLRGRFCGAKLPEPVVSTDSRLWVEFRS<br>KIILNFTSMDLYRSRLCWYDYVEVRDGFWRKAPLRGRFCGAKLPEPVVSTDSRLWVEFRS	361<br>420
H. BMP1<br>D. BMP1	SSNWVGKGFFAVYEAICGGDVKKDNGHIQSPNYPDDYRPSKVCVWRIQVSEGFHVGLTFQ<br>SSNWVGKGFFAVYEAICGGDVKKDNGHIQSPNYPDDYRPSKVCVWRIQVSEGFHVGLTFQ	421<br>480
H. BMP1<br>D. BMP1	SFEIERHDSCAYDYLEVRDGHSEGSPLIGRYCGYERPDDIKSTSSRLWLKFVSDGSINKA<br>SFEIERHDSCAYDYLEVRDGHSEGSPLIGRYCGYERPDDIKSTSSRLWLKFVSDGSINKA	481<br>540
H. BMP1<br>D. BMP1	GFAVNFFKEVDECSRPNRGGCEQRCLNTLGSYKCSCDPGYELAPDKRRCEAACGGFLTKL<br>GFAVNFFKEVDECSRPNRGGCEQRCLNTLGSYKCSCDPGYELAPDKRRCEAACGGFLTKL	541<br>600
H. BMP1<br>D. BMP1	NGSITSPGWPKEYPPNKNCIWQLVAPTQYRISLQFDFFETEGNDVCKYDFVEVRSGLTAD<br>NGSITSPGWPKEYPPNKNCIWQLVAPTQYRISLQFDFFETEGNDVCKYDFVEVRSGLTAD	601<br>660
H. BMP1<br>D. BMP1	SKLHGKFCGSEKPEVITSQYNNMRVEFKSDNTVSKKGFKAHFFSDKDECSKDNGGCQQDC<br>SKLHGKFCGSEKPEVITSQYNNMRVEFKSDNTVSKKGFKAHFFSDKDECSKDNGGCQQDC	661<br>714
H. BMP1<br>D. BMP1	VNTFGSYECQCRSGFVLHDNKHDCKEAGCDHKVTSTSGTITSPNWPDKYPGKKECTWAIS<br>VNTFGSYECQCRSGFILHDNKHDCKEAGCDHKVTSTSGTITSPNWPDKYPSKKECTWAIS	720<br>780
H. BMP1<br>D. BMP1	STPGHRVKLTFTEMDIESQPECAYDHLEVYDGRDAKADILGRFCGSKKPEPILATGSRMF<br>STPGHRVKLTFTEMDIESQPECAYDHLEVYDGRDAKADILGRFCGSKKPEPILATGSRMF	781<br>820
H. BMP1<br>D. BMP1	LRFYSDNSVQRKGFQASHSTECGGQVQAEVKTKDLYSHAQFGDNNYPGGVDCEWVIVAEE<br>LRFYSDNSVQRKGFQASHSTECGGQVQAEVKTKDLYSHAQFGDNNYPGGVDCEWVIVAEE	821<br>880
H. BMP1<br>D. BMP1	GYGVELVFQTFEVEEETDCGYDYMELFDGYDSTAPRLGRYCGSGPPEEVYSAGDSVLVKF<br>GYGVELVFQTFEVEEETDCGYDYMELFDGYDSTAPRLGRYCGSGPPEEVYSAGDSVLVKF	881<br>940

H. BMP1	HSDDTITKKGFHLRYTSTKFQDTLHSRK	941
D. BMP1	HSDDTITKKGFHLRYTSTKFQDTLHSRK	1020

Table S23. Comparative amino acid analysis of donkey and horse BMP2 gene

H. BMP2	MVAGTHCLLALLLPQVLLGGAAGLIPELGRRKFAASTGRSSSQPSDDVLSEFELRLLSMF	1
D. BMP2	MVAGTHCLLALLLPQVLLGGAAGLIPELGRRKFAASTGHSSSQPSDDVLSEFELRLLSMF	60
H. BMP2	GLKQRPTPSRDAVVPPYMLDLYRRHSGQPDAPAPDHRLERAASLANTVRSFHHEESLEEL	61
D. BMP2	GLKQRPTPSRDAVVPPYMLDLYRRHSGQPDAPAPDHRLERAASLANTVRSFHHEESLEEL	120
H. BMP2	PEMSGKTTRRFFFNLTSIPTEEFITSAELQVFREQMQDPWENNSNFHHRINIYEIIKPAT	121
D. BMP2	PEMSGKTTRRFFFNLTSIPTEEFITSAELQVFREQMQDPWENNSNFHHRINIYEIIKPAT	180
H. BMP2	ANSKFPVTRLLDTRLVNQNASRWERFDVTPAVMRWTAQGLANHGFVVEVAHLEENRGASK	181
D. BMP2	ANSKFPVTRLLDTRLVNQNASRWERFDVTPAVMRWTAQGLANHGFVVEVAHLEENRGASK	240
H. BMP2	RHVRISRSLHQDEHSWSQIRPLLVTFGHDGKGHPLHKREKRQAKHKQRKRLKSSCKRHPL	241
D. BMP2	RHVRISRSLHQDEHSWSQIRPLLVTFGHDGKGHPLHKREKRQAKHKQRKRLKSSCKRHPL	300
H. BMP2	YVDFSDVGWNDWIVAPPGYHAFYCHGECPFPLADHLNSTNHAIVQTLVNSVNSKIPKACC	301
D. BMP2	YVDFSDVGWNDWIVAPPGYHAFYCHGECPFPLADHLNSTNHAIVQTLVNSVNSKIPKACC	360
H. BMP2	VPTELSAISMLYLDENEKVVLKNYQDMVVEGCGCR	361
D. BMP2	VPTELSAISMLYLDENEKVVLKNYQDMVVEGCGCR	420

Table S24**.** Comparative amino acid analysis of donkey and horse BMP3 gene

H. BMP3<br>D. BMP3	MAGARRLLYLWLGCFCVSLAQGERLKQHFPELPKTVPGDRTAGGGSGPVLRPHDKVSEHM<br>MAGARRLLYLWLGCFCVSLAQGERLKQHFPELPKTVPGDRTAGGGSGPVLRPHDKVSEHM	1<br>60
H. BMP3<br>D. BMP3	LRLYDRYSGSGRAEAARTPGISERGSQSLRPQPLREGNTVRSFRAGAAGTLESKGLHIFN<br>LRLYDRYSGSGRAEAARTPGISERGSQSLRPQPLREGNTVRSFRAGAAGTLESKGLHIFN	61<br>120
H. BMP3<br>D. BMP3	LTSLTKSENILSATLYFYIGELINTSLSCPVSQGCSHHAQRKHIQIDLSAWILKSNRNQS<br>LTSLTKSENILSATLYFYIGELINTSLSCPVSQGCSHHAQRKHIQIDLSAWILKSNRNQS	121<br>180
H. BMP3<br>D. BMP3	QLLGNLSVDVAKPHRDFVSWLSKDITQFLRKAKENEEFLIGFNITSKGHQLPKKMLPFPE<br>QLLGNLSVDVAKPHRDFVSWLSKDITQFLRKAKENEEFLIGFNITSKGHQLPKKMLPFPE	181<br>240
H. BMP3<br>D. BMP3	PYILVYANDAAISEPENVVSSLQGHRNFPIGAVPKLDSHIRAALSIERRKKRSTGVLLPL<br>PYILVYANDAAISEPENVVSSLQGHRNFPIGAVPKLDSHIRAALSIERRKKRSTGVLLPL	241<br>300
H. BMP3<br>D. BMP3	QNNELPGAEYQYKEEGVWEERKPYKTLQTQPPEKSKNKKKQRKGPQQKSQTLQFDEQTLK<br>QNNELPGAEYQYKEEGVWEERKPYKTLQTQPPEKSKNKKKQRKGPQQKSQTLQFDEQTLK	301<br>360
H. BMP3<br>D. BMP3	KARRKQWIEPRNCARRYLKVDFADIGWSEWIISPKSFDAYYCSGACQFPMPKSLKPSNHA<br>KARRKQWIEPRNCARRYLKVDFADIGWSEWIISPKSFDAYYCSGACQFPMPKSLKPSNHA	361<br>420
H. BMP3<br>D. BMP3	TIQSIVRAVGVVPGIPEPCCVPEKMSSLSILFFDENKNVVLKVYPNMTVESCACR<br>TIQSIVRAVGVVPGIPEPCCVPEKMSSLSILFFDENKNVVLKVYPNMTVESCACR	421<br>480

Table S25. Comparative amino acid analysis of donkey and horse BMP4 gene

H. BMP4	MIPGNRMLMVVLLCQVLLGGASHASLIPETGKKKVAEIQGHAGGRRSGQSHELLRDFEAT	1
D. BMP4	MIPGNRMLMVVLLCQVLLGGASHASLIPETGKKKVAEIQGHAGGRRSGQSHELLRDFEAT	60
H. BMP4	LLQMFGLRRRPQPSKNAVVPDYMRDLYRLQSGEEEEEEQIHSVGLEYPERPASRANTVRS	61
D. BMP4	LLQMFGLRRRPQPSKSAVVPDYMRDLYRLQSGEEEEEEQIHSVGLEYPERPASRANTVRS	120
H. BMP4	FHHEEHLESIPGTSENSAFRFLFNLSSIPENEVISSAELRLFREQVDQGPDWEQGFHRIN	121
D. BMP4	FHHEEHLESIPGTSENSAFRFLFNLSSIPENEVISSAELRLFREQVDQGPDWEQGFHRIN	180
H. BMP4	VYEVMKPPAEVVPGRLITRLLDTRLVHHSVTRWETFDVSPAVLRWTREKQPNYGLAIEVT	181
D. BMP4	VYEVMKPPAEVVPGHLITRLLDTRLVHHNVTRWETFDVSPAVLRWTREKQPNYGLAIEVT	240
H. BMP4	PLHQTRTHQGQHVRISRALPQGSGDWAQLRPLLVTFGHDGRGHALTRRRRAKRSPKHHPQ	241
D. BMP4	HLHQTRTHQGQHVRISRSLPQGSGDWAQLRPLLVTFGHDGRGHALTRRRRAKRSPKHHPQ	300
H. BMP4	RARKKTKNCRRHSLYVDFSDVGWNDWIVAPPGYQAFYCHGDCPFPLADHLNSTNHAIVQT	301
D. BMP4	RARKKTKNCRRHSLYVDFSDVGWNDWIVAPPGYQAFYCHGDCPFPLADHLNSTNHAIVQT	360
H. BMP4	LVNSVNSSIPKACCVPTELSAISMLYLDEYDKVVLKNYQEMVVEGCGCR	361
D. BMP4	LVNSVNSSIPKACCVPTELSAISMLYLDEYDKVVLKNYQEMVVEGCGCR	420

Table S26. Comparative amino acid analysis of donkey and horse BMP5 gene

H. BMP5<br>D. BMP5	MHLTVFLLRGIVGFLWSCWVLVGYAKGGLGDNHVHSSFIYRRLRNHERREIQREILSILG<br>MHLTVFLLRGIVGFLWSCWVLVGYAKGGLGDNHVHSSFIYRRLRNHERREIQREILSILG	1<br>60
H. BMP5<br>D. BMP5	LPHRPRPFSPGKQASSAPLFMLDLYNAMANEENPDETEYSVRASLAGETRGGRKGYPASP<br>LPHRPRPFSPGKQASSAPLFMLDLYNAMANEENPDETEYSVRASLAGETRGGRKGYPASP	61<br>120
H. BMP5<br>D. BMP5	NGYPRGIQLSRTAPLTTQSPPLASLHDTNFLNDADMVMSFVNLVERDKDFSHQRRHYKEF<br>NGYPRGIQLSRTAPLTTQSPPLASLHDTNFLNDADMVMSFVNLVERDKDFSHQRRHYKEF	121<br>180
H. BMP5<br>D. BMP5	RFDLTQIPHGEAVTAAEFRIYKDRSNSRFENETIKISIYQIIKEYTNRDADLFLLDTRKA<br>RFDLTQIPHGEAVTAAEFRIYKDRSNSRFENETIKISIYQIIKEYTNRDADLFLLDTRKA	181<br>240
H. BMP5<br>D. BMP5	EALDVGWLVFDITVTSNHWVINPQNNLGLQLCAETGDGRSINVKSAGLVGRHGPQSKQPF<br>EALDVGWLVFDITVTSNHWVINPQNNLGLQLCAETGDGRSINVKSAGLVGRHGPQSKQPF	241<br>300
H. BMP5<br>D. BMP5	MVAFFKASEVLLRSVRAANKRKNQNRNKSSSHQDSSRVSSVGDYNTSEQKQACKKHELYV<br>MVAFFKASEVLLRSVRAANKRKNQNRNKSSSHQDSSRVSSVGDYNTSEQKQACKKHELYV	301<br>360
H. BMP5<br>D. BMP5	SFRDLGWQDWIIAPEGYAAFYCDGECSFPLNAHMNATNHAIVQTLVHLMFPDHVPKPCCA<br>SFRDLGWQDWIIAPEGYAAFYCDGECSFPLNAHMNATNHAIVQTLVHLMFPDHVPKPCCA	361<br>420
H. BMP5<br>D. BMP5	PTKLNAISVLYFDDSSNVILKKYRNMVVRSCGCH<br>PTKLNAISVLYFDDSSNVILKKYRNMVVRSCGCH	421<br>480

Table S27**.** Comparative amino acid analysis of donkey and horse BMP6 gene

H. BMP6<br>D. BMP6	MPGLARRAQWLCWWWGLLCSCCGPPPLRPSLPAAAATGGGALLGDGGSPGHAEQPPPPPQ<br>MPGLARRAQWLCWWWGLLCSCCGPPPLRPSLPAAAATGGGALLGDGGSPGHAEQPPPPPQ	1<br>60
H. BMP6<br>D. BMP6	SSSSGFLYRRLKTHEKREMQKEILSVLGLPHRPRPLHGLQQLQPPALPQQQPRGEPPPGR<br>SSSSGFLYRRLKTHEKREMQKEILSVLGLPHRPRPLHGLQQLQPPALPQQQPRGEPPPGR	61<br>120
H. BMP6<br>D. BMP6	LKSAPLFMLDLYNALAAADDEDGPSDEERRPPAPRGGAGSPQPGQPPPGAAHPLNRKSLL<br>LKSAPLFMLDLYNALAAADDEDGPSDEERRPPAPRGGAGSPQPGQPPPGAAHPLNRKSLL	121<br>180
H. BMP6<br>D. BMP6	APGPGGGGAATPLTSAQDSAFLNDADMVMSFVNLVEYDKEFSPRQRHHKEFKFNLSQIPE<br>APGPGGGGAATPLTSAQDSAFLNDADMVMSFVNLVEYDKEFSPRQRHHKEFKFNLSQIPE	181<br>240
H. BMP6<br>D. BMP6	GEAVTAAEFRIYKDCVVGSFKNQTFLISIYQVLQEHQHRDSDLFLLDTRMVWASEEGWLE<br>GEAVTAAEFRIYKDCVVGSFKNQTFLISIYQVLQEHQHRDSDLFLLDTRMVWASEEGWLE	241<br>300
H. BMP6<br>D. BMP6	FDITATSNLWVVTPQHNMGLQLSVVTRDGLSINPRAAGLVGRDGPYDKQPFMVAFFKVSE<br>FDITATSNLWVVTPQHNMGLQLSVVTRDGLSINPRAAGLVGRDGPYDKQPFMVAFFKVSE	301<br>360
H. BMP6<br>D. BMP6	VHVRTTRSATGRRRQQSRNRSTQSQDVSRVSSASDYNSSELKTACRKHELYVSFQDLGWQ<br>VHVRTTRSATGRRRQQSRNRSTQSQDVSRVSSASDYNSSELKTACRKHELYVSFQDLGWQ	361<br>420
H. BMP6<br>D. BMP6	DWIIAPKGYAANYCDGECSFPLNAHMNATNHAIVQTLVHLMNPEYVPKPCCAPTKLNAIS<br>DWIIAPKGYAANYCDGECSFPLNAHMNATNHAIVQTLVHLMNPEYVPKPCCAPTKLNAIS	421<br>480
H. BMP6<br>D. BMP6	VLYFDDNSNVILKKYRNMVVRACGCH<br>VLYFDDNSNVILKKYRNMVVRACGCH	481<br>540

Table S28. Comparative amino acid analysis of donkey and horse BMP7 gene

H. BMP7	MHVRSLRTAAPHSFVALWAPLFLLRSALADFSLDNEVHSSFIHRRLRSQERREMQREILS	1
D. BMP7	MHVRSLRTAAPHSFVAFWAPLFLLRSALADFSLDNEVHSSFIHRRLRSQERREMQREILS	60
H. BMP7	ILGLPHRPRPHLQGKHNSAPMFMLDLYNAMAVEESGGPDGQGFSYPHKAVSSTQGPPLAS	61
D. BMP7	ILGLPHRPRPHLQGKHNSAPMFMLDLYNAMAVEESGGPDGQGFSYPHKAVFSTQGPPLAS	120
H. BMP7	LQDSHFLTDADMVMSFVNLVEHDKEFFHPRYHHREFRFDLSKIPEGEAVTAAEFRIYKDY	121
D. BMP7	LQDSHFLTDADMVMSFVNLVEHDKEFFHPRYHHREFRFDLSKIPEGEAVTAAEFRIYKDY	180
H. BMP7	VRERFDNETFRISVYQVLQEHLARESDLFLLDSRTLWASEEGWLVFDITATSNHWVVNPR	181
D. BMP7	VRERFDNETFRISVYQVLQEHLARESDLFLLDSRTLWASEEGWLVFDITATSNHWVVNPR	240
H. BMP7	HNLGLQLSVETLDGQSVNPKLAGLIGRHGPQTKQPFMVAFFKATEVHLRSTRSTGGKQRS	241
D. BMP7	HNLGLQLSVETLDGQSVNPKLAGLIGRHGPQTKQPFMVAFFKATEVHLRSTRSTGGKQRS	300
H. BMP7	QNRSKTPKNQEALRVANVAENSSSDQRQACKKHELYVSFRDLGWQDWIIAPEGYAAYYCE	301
D. BMP7	QNRSKTPKNQEALRVANVAENSSSDQRQACKKHELYVSFRDLGWQDWIIAPEGYAAYYCE	360
H. BMP7	GECAFPLNSYMNATNHAIVQTLVHFINPETVPKPCCAPTQLNAISVLYFDDSSNVILKKY	361
D. BMP7	GECAFPLNSYMNATNHAIVQTLVHFINPETVPKPCCAPTQLNAISVLYFDDSSNVILKKY	420
H. BMP7	RNMVVRACGCH	421
D. BMP7	RNMVVRACGCH	480

Table S29. Comparative amino acid analysis of donkey and horse BMP10 gene

H.BMP10<br>D.BMP10	MGSLALELCALFCLVAHLVSGSPIMSLERSPLEEDMPLFDDVFSEQDGVDFNTLLQSMKN<br>MGSLALELCALFCLVAHLVSGSPIMSLERSPLEEDMPLFDDVFSEQDGVDFNTLLQSMKN	1<br>60
H.BMP10<br>D.BMP10	EFLKTLNLSDIPMQDSAKVDPPEYMLELYNKFATDRTSMPSANIIRSFKNEDLFSQPASF<br>EFLKTLNLSDIPMQDSAKVDPPEYMLELYNKFATDRTSMPSANIIRSFKNEDLFSQPASF	61<br>120
H.BMP10<br>D.BMP10	NGLRKYPLFFNVSIPHHEEVIMAELRLYTLVQRDRIIYDGVDRKITIYEVLESKGDNEGE<br>NGLRKYPLFFNVSIPHHEEVIMAELRLYTLVQRDRIIYDGVDRKITIYEVLESKGDNEGE	121<br>180
H.BMP10<br>D.BMP10	RTMLVLVSGEIYGTNSEWETFDVTNAIRHWQKSGLSTHQLEVHIESRQDEAEDAGRGQLE<br>RTMLVLVSGEIYGTNSEWETFDVTNAIRHWQKSGLSTHQLEVHIESRQDEAEDAGRGQLE	181<br>240
H.BMP10<br>D.BMP10	IDTSAENKHDPLLVVFSDDQSSEKEGKEELTEMIAHEQLLELDNLGLEGFSSGPGEEALL<br>IDTSAENKHDPLLVVFSDDQSSEKEGKEELSEMIAHEQLLELDNLGLDGFSSGPGEEALL	241<br>300
H.BMP10<br>D.BMP10	QMRSNIIYDSTARIRRNAKGNYCKRTPLYIDFKEIGWDSWIIAPPGYEAYECRGVCNYPL<br>QMRSNIIYDSTARIRRNAKGNYCKRTPLYIDFKEIGWDSWIIAPPGYEAYECRGVCNYPL	301<br>360
H.BMP10<br>D.BMP10	AEHLTPTKHAIIQALVHLKNSQKASKACCVPTKLEPISILYLDKGVVTYKFKYEGMAVSE<br>AEHLTPTKHAIIQALVHLKNSQKASKACCVPTKLEPISILYLDKGVVTYKFKYEGMAVSE	361<br>420
H.BMP10<br>D.BMP10	CGCR<br>CGCR	421<br>480

Table S30. Comparative amino acid analysis of donkey and horse BMP15 gene

H.BMP15<br>D.BMP15	MVLLSILRILLWGLVLFREHRVQMAKVGQPSIALPAEVPTLPLILELLEEAPAKQQGKPQ<br>MVLLSILRILLWGLVLYREHRVQMAKVGQPSIALPAEVPTLPLILELLEEAPAKQQGKPQ	1<br>60
H.BMP15<br>D.BMP15	VLGHPLRYMLELYQRSADAHGHPRENRTIGATMVRLVKPLTNVARPLRGPWHIQTLDFPL<br>VLGHPLRYMLELYQRSADAHGHPRENRTIGATMVRLVKPLTNVARPLRGPWHIQTLDFPL	61<br>120
H.BMP15<br>D.BMP15	RSNRVKYQLVRATVVYRHQLHLSHFNLSCYVEPWVQKSPTNQFPSSGRVSSKPSLLSKAW<br>RSNRVKYQLVRATVVYRHQLHLSHFNLSCHVEPWVQKSPTNQFPSSGRVSSKPSLLSKAW	121<br>180
H.BMP15<br>D.BMP15	TEMDITQHIRQRLWNHKGRRVLRLRFVCQQPKDSEVLELRWHGTSSLDTVFLLLYFNDTH<br>TEMDITQHIRQRLWNHKGRRVLRLRFVCQQPKDSEVLELRWHGTSSLDTVFLLLYFNDTH	181<br>240
H.BMP15<br>D.BMP15	KSGQKTKLLPRGLEEFMERDASLLLRRVRQAGSMGSEVLGPSREREGPESNQCSLHPFQV<br>KSGQKTKLLPRGLEEFMERDASLLLRRVRQAGSMGSEVLGPSREREGPESNQCSLHPFQV	241<br>300
H.BMP15<br>D.BMP15	SFHQLGWDHWIIAPHLYTPNYCKGACPRVLRYGLNSPNHAIIQSLVNELVDQSVPPPSCV<br>SFHQLGWDHWIIAPHLYTPNYCKGACPRVLRYGLNSPNHAIIQSLVNELVDQSVPPPSCV	301<br>360
H.BMP15<br>D.BMP15	PYKYVPISLLLIEANGSILYKEYENMIAQSCTCR<br>PYKYVPISLLLIEANGSILYKEYENMIAQSCTCR	361<br>420

Table S31. Comparative amino acid analysis of donkey and horse BMP8B gene

H.BMP8B<br>D.BMP8B	MAARPSPLWLLGLALCALGGGGPGPRPPPGCPPRRLGPRERRDLQREILEVLGLPGRPRP<br>MAARPGPLWLLGLALCALGGGGPGPRPPPGCPPRRLGPRERRDLQREILEVLGLPGRPRP	1<br>60
H.BMP8B<br>D.BMP8B	RTPPAAARPPASAPLFMLDLYHAMAGDDAEDGGPPERRLGHADLVMSFVNMVELDRTLGH<br>RTPPAAARPPASAPLFMLDLYHAMAGDDAEDGGPPERRLGHADLVMSFVNMVELDRTLGH	61<br>120
H.BMP8B<br>D.BMP8B	QEPHWKEFRFDLTQIPAGEAVTAAEFRIYKLPSTHPLNRTLHVSMFEVVRERANRESDLF<br>QEPHWKEFRFDLTQIPAGEAITAAEFRIYKLPSTHPLNRTLHVSMFEVVRERANRESDLF	121<br>180
H.BMP8B<br>D.BMP8B	FLDLQTLRAGDEGWLVLDVTAASDRWLLNRNKDLGLRLYVETEDGAPDEARRCPQGVLRA<br>FLDLQTLQAGDEGWLVLDVTAASDRWLLNRNKDLGLRLYVETEDGAPDEARRCPQGVLRA	181<br>240
H.BMP8B<br>D.BMP8B	HQAERHLRALLRQQQQCHPAQAPQHGGPRLRLPLRAPRPLGASPSSGPSAEAGAPQTQPA<br>HQAERHLRALLRQQQQRHPAQAPQHGGPRLRLPLRAPRPLGASPSSGSSAEAGAPQTQPA	241<br>300
H.BMP8B<br>D.BMP8B	PGGVGTEGPPRSTCLCPCLPPARLGPLQAPPPLCPV<br>PGGVGTEGPPRSTCLCPCLPPARLGPLQAPPPLCPV	301<br>360

Table S32. Comparative amino acid analysis of donkey and horse BMPR1A gene

H.BMPR1A<br>D.BMPR1A	MTQLSIYIRLLGAYLFIISHVQGQNLDSMLHGTGVKSDPEQKKSENGATVAPEDTLPFLK<br>MTQLYIYIRLLGAYLFIISHVQGQNLDSMLHGTGVKSDPEQKKSENGATVAPEDTLPFLK	1<br>60
H.BMPR1A<br>D.BMPR1A	CYCSGHCPDDAINNTCITNGHCFAIIEEDDQGETTLASGCMKYEGSDFQCKDSPKAQLRR<br>CYCSGHCPDDAINNTCITNGHCFAIIEEDDQGETTLASGCMKYEGSDFQCKDSPKAQLRR	61<br>120
H.BMPR1A<br>D.BMPR1A	TIECCRTNLCNQYLQPTLPPVVIGPFFDGSIRWLVVLISMAVCIIAMIIFSSCFCYKHYC<br>TIECCRTNLCNQYLQPTLPPVVIGPFFDGSIRWLVVLISMAVCIIAMIIFSSCFCYKHYC	121<br>180
H.BMPR1A<br>D.BMPR1A	KSISSRRRYNRDLEQDEAFIPVGESLKDLIDQSQSSGSGSGLPLLVQRTIAKQIQMVRQV<br>KSISSRRRYNRDLEQDEAFIPVGESLKDLIDQSQSSGSGSGLPLLVQRTIAKQIQMVRQV	181<br>240
H.BMPR1A<br>D.BMPR1A	GKGRYGEVWMGKWRGEKVAVKVFFTTEEASWFRETEIYQTVLMRHENILGFIAADIKGTG<br>GKGRYGEVWMGKWRGEKVAVKVFFTTEEASWFRETEIYQTVLMRHENILGFIAADIKGTG	241<br>300
H.BMPR1A<br>D.BMPR1A	SWTQLYLITDYHENGSLYDFLKCATLDTRALLKLAYSAACGLCHLHTEIYGTQGKPAIAH<br>SWTQLYLITDYHENGSLYDFLKCATLDTRALLKLAYSAACGLCHLHTEIYGTQGKPAIAH	301<br>360
H.BMPR1A<br>D.BMPR1A	RDLKSKNILIKKNGSCCIADLGLAVKFNSDTNEVDVPLNTRVGTKRYMAPEVLDESLNKN<br>RDLKSKNILIKKNGSCCIADLGLAVKFNSDTNEVDVPLNTRVGTKRYMAPEVLDESLNKN	361<br>420
H.BMPR1A<br>D.BMPR1A	HFQPYIMADIYSFGLIIWEMARRCVTGGIVEEYQLPYYNMVPNDPSYEDMREVVCVKRLR<br>HFQPYIMADIYSFGLIIWEMARRCVTGGIVEEYQLPYYNMVPNDPSYEDMREVVCVKRLR	421<br>480
H.BMPR1A<br>D.BMPR1A	PLVSNRWNSDECLRAVLKLMSECWAHNPASRLTALRIKKTLAKMVESQDVKI<br>PLVSNRWNSDECLRAVLKLMSECWAHNPASRLTALRIKKTLAKMVESQDVKI	481<br>540

Table S33. Comparative amino acid analysis of donkey and horse BMPR1B gene

H.BMPR1B	MQQISAAYLSANFLDNMLLRSSGKLNVGTKKEDGESASPTPRPKILRCKCHHHCPEDSVN	1
D.BMPR1B	MQQISAAYLSANFLDNMLLRSSGKLNVGTKKEDGESASPTPRPKILRCKCHHHCPEDSVN	60
H.BMPR1B	NICSTDGYCFTMIEEDDSGMPVVTSGCLGLEGSDFQCRDTPIPHQRRSIECCTERNECNE	61
D.BMPR1B	NICSTDGYCFTMIEEDDSGMPVVTSGCLGLEGSDFQCRDTPIPHQRRSIECCTERNECNE	120
H.BMPR1B	DLHPTLPPLKTRDFVDGPIHHKALLISVTVCSLLLVLIIILFCYFRYKRQETRPRYSIGLE	121
D.BMPR1B	DLHPTLPPLKTRDFVDGPIHHKALLISVTVCSLLLVLIIILFCYFRYKRQETRPRYSIGLE	180
H.BMPR1B	QDETYIPPGESLRDLIEQSQSSGSGSGLPLLVQRTIAKQIQMVKQIGKGRYGEVWMGKWR	181
D.BMPR1B	QDETYIPPGESLRDLIEQSQSSGSGSGLPLLVQRTIAKQIQMVKQIGKGRYGEVWMGKWR	240
H.BMPR1B	GEKVAVKVFFTTEEASWFRETEIYQTVLMRHENILGFIAADIKGTGSWTQLYLITDYHEN	241
D.BMPR1B	GEKVAVKVFFTTEEASWFRETEIYQTVLMRHENILGFIAADIKGTGSWTQLYLITDYHEN	300
H.BMPR1B	GSLYDYLKSTTLDTKSMLKLAYSSVSGLCHLHTEIFSTQGKPAIAHRDLKSKNILVKKNG	301
D.BMPR1B	GSLYDYLKSTTLDTKSMLKLAYSSVSGLCHLHTEIFSTQGKPAIAHRDLKSKNILVKKNG	360
H.BMPR1B	TCCIADLGLAVKFISDTNEVDIPPNTRVGTKRYMPPEVLDESLNRNHFQSYIMADMYSFG	361
D.BMPR1B	TCCIADLGLAVKFISDTNEVDIPPNTRVGTKRYMPPEVLDESLNRNHFQSYIMADMYSFG	420
H.BMPR1B	LILWEVARRCVSGGIVEEYQLPYHDLVPSDPSYEDMREIVCLKKLRPSFPNRWTSDECLR	421
D.BMPR1B	LILWEVARRCVSGGIVEEYQLPYHDLVPSDPSYEDMREIVCIKKLRPSFPNRWTSDECLR	480
H.BMPR1B	QMGKLMTECWAHNPASRLTALRVKKTLAKMSESQDIKL	481
D.BMPR1B	QMGKLMTECWAHNPASRLTALRVKKTLAKMSESQDIKL	540

Table S34. Comparative amino acid analysis of donkey and horse BRINP1 gene

H.BRINP1<br>D.BRINP1	MNWRFVELLYFLFIWGRISVQPSHQEPAGTDQHVSKEFDWLISDRGPFHHSRSYLSFVER<br>MNWRFVELLYFLFIWGRISVQPSHQEPAGTDQHVSKEFDWLISDRGPFHHSRSYLSFVER	1<br>60
H.BRINP1<br>D.BRINP1	HRQGFTTRYKIYREFARWKVRNTAIERRDLVRHPLPLMPEFQRSIRLLGRRPTTQQFIDT<br>HRQGFTTRYKIYREFARWKVRNTAIERRDLVRHPLPLMPEFQRSIRLLGRRPTTQQFIDT	61<br>120
H.BRINP1<br>D.BRINP1	IIKKYGTHLLISATLGGEEALTMYMDKSRLDRKSGNATQSVEALHQLASSYFVDRDGTMR<br>IIKKYGTHLLISATLGGEEALTMYMDKSRLDRKSGNATQSVEALHQLASSYFVDRDGTMR	121<br>180
H.BRINP1<br>D.BRINP1	RLHEIQISTGAIKVTETRTGPLGCNSYDNLDSVSSVLLQSTESKLHLQGLQIIFPQYLQE<br>RLHEIQISTGAIKVTETRTGPLGCNSYDNLDSVSSVLLQSTESKLHLQGLQIIFPQYLQE	181<br>240
H.BRINP1<br>D.BRINP1	KFVQSALSYIMCNGEGEYVCQNSQCRCQCAEEFPQCNCPITDIQIMEYTLANMAKSWAEA<br>KFVQSALSYIMCNGEGEYVCQNSQCRCQCAEEFPQCNCPITDIQIMEYTLANMAKSWAEA	241<br>300
H.BRINP1<br>D.BRINP1	YKDLENSDEFKSFMKRLPSNHFLTIGSIHQHWGNDWDLQNRYKLLQSATEAQRQKIQRTA<br>YKDLENSDEFKSFMKRLPSNHFLTIGSIHQHWGNDWDLQNRYKLLQSATEAQRQKIQRTA	301<br>360
H.BRINP1<br>D.BRINP1	RKLFGLSVRCRHNPNHQLPRERTIQQWLARVQSLLYCNENGFWGTFLESQRSCVCHGSTT<br>RKLFGLSVRCRHNPNHQLPRERTIQQWLARVQSLLYCNENGFWGTFLESQRSCVCHGSTT	361<br>420
H.BRINP1<br>D.BRINP1	LCQRPIPCIIGGNNSCAMCSLANISLCGSCNKGYKLYRGRCEPQNVDSERSEQFISFETD<br>LCQRPIPCIIGGNNSCAMCSLANISLCGSCNKGYKLYRGRCEPQNVDSERSEQFISFETD	421<br>480
H.BRINP1<br>D.BRINP1	LDFQDLELKYLLQKMDSRLYVHTTFISNEIRLDTFFDPRWRKRMSLTLKSNKNRMDFIHM<br>LDFQDLELKYLLQKMDSRLYVHTTFISNEIRLDTFFDPRWRKRMSLTLKSNKNRMDFIHM	481<br>540
H.BRINP1<br>D.BRINP1	VIGMSMRICQMRNSSLDPMFFVYVNPFSGSHSEGWNMPFGEFGYPRWEKIRLQNSQCYNW<br>VIGMSMRICQMRNSSLDPMFFVYVNPFSGSHSEGWNMPFGEFGYPRWEKIRLQNSQCYNW	541<br>600
H.BRINP1<br>D.BRINP1	TLLLGNRWKTFFETVHIYLRSRTRLPTLLRNETGQGPVDLSDPSKRQFYIKISDVQVFGY<br>TLLLGNRWKTFFETVHIYLRSRTRLPTLLRNETGQGPVDLSDPSKRQFYIKISDVQVFGY	601<br>660
H.BRINP1<br>D.BRINP1	SCMLKHRLKLTNSEIIRVNHALDLYNTEILKQSDQMTAKLC<br>SCMLKHRLKLTNSEIIRVNHALDLYNTEILKQSDQMTAKLC	661<br>714

Table S35. Comparative amino acid analysis of donkey and horse BRINP2 gene

H.BRINP2<br>D.BRINP2	MRWPCGTRFRGLRLAAAPWAALLALGLPGWVLAVSASAAAVVPEQHASSAGQPPLDWLLT<br>MRWPCGTRFRGLRLAAAPWAALLALGLPGWVLAVSASAAAVVPEQHASSAGQPPLDWLLT	1<br>60
H.BRINP2<br>D.BRINP2	DRGPFHRAQEYADFMERYRQGFTTRYRIYREFARWKVNNLVLERKDFFSLPLPLAPEFIR<br>DRGPFHRAQEYADFMERYRQGFTTRYRIYREFARWKVNNLVLERKDFFSLPLPLAPEFIR	61<br>120
H.BRINP2<br>D.BRINP2	NIRLLGRRPNLQQVTENLIKKYGTHFLLSATLGGEESLTIFVDKRKLSRKTETAGGAPVV<br>NIRLLGRRPNLQQVTENLIKKYGTHFLLSATLGGEESLTIFVDKRKLSRKTETAGGAPVV	121<br>180
H.BRINP2<br>D.BRINP2	GGSGNSSAVSLEILHQLAASYFIDRESTLRRLHHIQIATGAIKVSETRTGPLGCSNYDNL<br>GGSGNSSAVSLEILHQLAASYFIDRESTLRRLHHIQIATGAIKVSETRTGPLGCSNYDNL	181<br>240
H.BRINP2<br>D.BRINP2	DSVSSVLVQSPENKVQLLGLQVLLPEYLRERFVAAALSYITCSSEGELVCKGNDCWCKCS<br>DSVSSVLVQSPENKVQLLGLQVLLPEYLRERFVAAALSYITCSSEGELVCKGNDCWCKCS	241<br>300
H.BRINP2<br>D.BRINP2	PTFPECNCPDTDIQAMEDSLLQIQDSWATHNRQFEESEEFQALLKRLPDDRFLNSTAISQ<br>PTFPECNCPDTDIQAMEDSLLQIQDSWATHNRQFEESEEFQALLKRLPDDRFLNSTAISQ	301<br>360
H.BRINP2<br>D.BRINP2	FWAMDTGLQHRYQQLGASLKVLFKKTHRIVRRLFNLCKRCHRQPRFRLPKERSLSYWWNR<br>FWAMDTSLQHRYQQLGASLKVLFKKTHRIVRRLFNLCKRCHRQPRFRLPKERSLSYWWNR	361<br>420
H.BRINP2<br>D.BRINP2	IQSLLYCGESTFPGTFLEQSHSCTCPYDQSSCQGPIPCALGEGPACAHCAPDNSTRCGSC<br>IQSLLYCGESTFPGTFLEQSHSCTCPYDQSSCQGPIPCALGEGPACAHCAPDNSTRCGSC	421<br>480
H.BRINP2<br>D.BRINP2	NPGYVLAQGLCRPEVAESLENFLGLETDLQDLELKYLLQKRDSRIEVHSIFISNDMRLGS<br>NPGYVLAQGLCRPEVAESLENFLGLETDLQDLELKYLLQKRDSRIEVHSIFISNDMRLGS	481<br>540
H.BRINP2<br>D.BRINP2	WFDPSWRKRMLLTLKSNKYKPGLVHVMLALSLQICLTKNSTLEPVMAIYVNPFGGSHSES<br>WFDPSWRKRMLLTLKSNKYKPGLVHVMLALSLQICLTKNSTLEPVMAIYVNPFGGSHSES	541<br>600
H.BRINP2<br>D.BRINP2	WFMPVNEGNFPDWERTNVDAAAQCQNWTITLGNRWKTFFETVHVYLRSRIKSLDDSSNET<br>WFMPVNEGNFPDWERTNVDAAAQCQNWTITLGNRWKTFFETVHVYLRSRIKSLDDSSNET	601<br>660
H.BRINP2<br>D.BRINP2	IYYEPLEMTDPSKNLGYMKINTLQVFGYSLPFDPDAIRDLILQLDYPYTQGSQDSALLQL<br>IYYEPLEMTDPSKNLGYMKINTLQVFGYSLPFDPDAIRDLILQLDYPYTQGSQDSALLQL	661<br>714
H.BRINP2<br>D.BRINP2	IELRDRVNQLSPPGKVRLDLFSCLLRHRLKLANNEVGRIQSSLRAFNSKLPNPVEYETGK<br>IELRDRVNQLSPPGKVRLDLFSCLLRHRLKLANNEVGRIQSSLRAFNSKLPNPVEYETGK	601<br>660
H.BRINP2<br>D.BRINP2	LCS<br>LCS	661<br>714

Table S36. Comparative amino acid analysis of donkey and horse BRINP3 gene

H.BRINP3<br>D.BRINP3	EFGRWKVNNLAVERRNFLGSPLPLAPEFFRNIRLLGRRPTLQQITENLIKKYGTHFLLSA<br>EFGRWKVNNLAVERRNFLGSPLPLAPEFFRNIRLLGRRPTLQQITENLIKKYGTHFLLSA	1<br>60
H.BRINP3<br>D.BRINP3	TLGGEESLTIFVDKRKLSKRSEGSDSSTNSSSVTLETLHQLAASYFIDRDSTLRRLHHIQ<br>TLGGEESLTIFVDKRKLSKRSEGSDSSTNSSSVTLETLHQLAASYFIDRDSTLRRLHHIQ	61<br>120
H.BRINP3<br>D.BRINP3	IASTAIKVTETRTGPLGCSNYDNLDSVSSVLVQSPENKIQLQGLQVLLPDYLQERFVQAA<br>IASTAIKVTETRTGPLGCSNYDNLDSVSSVLVQSPENKIQLQGLQVLLPDYLQERFVQAA	121<br>180
H.BRINP3<br>D.BRINP3	LSYIACNSEGEFICKDNDCWCHCGPKFPECNCPSMDIQAMEENLLRITETWKAYNSDFEE<br>LSYIACNSEGEFICKDNDCWCHCGPKFPECNCPSMDIQAMEENLLRITETWKAYNSDFEE	181<br>240
H.BRINP3<br>D.BRINP3	SDEFKFFMKRLPMNYFLNTSTIMHLWTMDSNFQRRYEQLESSMKQLFLKAQKIVHKVFSL<br>SDEFKFFMKRLPMNYFLNTSTIMHLWTMDSNFQRRYEQLESSMKQLFLKAQKIVHKVFSL	241<br>300
H.BRINP3<br>D.BRINP3	SKRCHKQPLISLPRQRTSTYWLTRIQSFLYCNENGLLGSFSEETHSCTCPSDQVACTTPL<br>SKRCHKQPLISLPRQRTSTYWLTRIQSFLYCNENGLLGSFSEETHSCTCPSDQVACTTPL	301<br>360
H.BRINP3<br>D.BRINP3	PCAVGDAAACLSCAPDNRTRCGACNTGYMLSQGLCKPEVAEATDHYIGFETDLQDLEMKY<br>PCAVGDAAACLSCAPDNRTRCGACNTGYMLSQGLCKPEVAEATDHYIGFETDLQDLEMKY	361<br>420
H.BRINP3<br>D.BRINP3	LLQKTDRRIEVHAIFISNDMRLNSWFDPSWRKRMLLTLKSNKYKSSLVHMILGLSLQICL<br>LLQKTDRRIEVHAIFISNDMRLNSWFDPSWRKRMLLTLKSNKYKSSLVHMILGLSLQICL	421<br>480
H.BRINP3<br>D.BRINP3	TKNSTLEPVLAVYVNPFGGSHSESWFMPVNENSFPDWERTKLDLPLQCYNWSLTLGNKWK<br>TKNSTLEPVLAVYVNPFGGSHSESWFMPVNENSFPDWERTKLDLPLQCYNWSLTLGNKWK	481<br>540
H.BRINP3<br>D.BRINP3	TFFETVHIYLRSRIKSNGPNGNESIYYEPLEFIDPSRNLGYMKINNIQVFGYSMHFDPEA<br>TFFETVHIYLRSRIKSNGPNGNESIYYEPLEFIDPSRNLGYMKINNIQVFGYSMHFDPEA	541<br>600
H.BRINP3<br>D.BRINP3	IRDLILQLDYPYTQGSQDSALLQLLEIRDRVNKLSPPGQRRLDLFSCLLRHRLKLSTSEV<br>IRDLILQLDYPYTQGSQDSALLQLLEIRDRVNKLSPPGQRRLDLFSCLLRHRLKLSTSEV	601<br>660
H.BRINP3<br>D.BRINP3	VRILSALQAFNAKLPNTVDYDTTKLCS<br>VRILSALQAFNAKLPNTVDYDTTKLCS	661<br>714

Table S37**.** Comparative amino acid analysis of donkey and horse RGMA gene

H. RGMA<br>D. RGMA	MREGAVAAGRGQPSPHPHRLPRPLPPAPPALAPRPRPGRSARPLPSALKGTSRRRARRWR<br>MREGAVAAGRGQPSPHPHRLPRPLPPAPPALAPRPRPGRSARPLPSALKGTSRRRARRWR	1<br>60
H. RGMA<br>D. RGMA	AGAGRGRGGPSRPTLQLSPERGLRRPSRWVERHQPFAPGPAPPGARRHAAREQAACPGPL<br>AGAGRGRGGPSRPTLQLSPERGLRRPSRWVERHQPFAPGPAPPGARRHAAREQAACPGPL	61<br>120
H. RGMA<br>D. RGMA	LSRPGRRGRALVEPLPPHPGADRRRPGTPSSPAAPPAPPPAGRRGSCSRQATSPCKILKC<br>LSRPGRRGRALVEPLPPHPGADRRRPGTPSSPAAPPAPPSAGRRGSCSRQATSPCKILKC	121<br>180
H. RGMA<br>D. RGMA	NSEFWSATAGSQSQVADEAREFCAALRTYALCTRRTARTCRGDLAYHSAVHGIEDLMSQH<br>NSEFWSATAGSQSQVADEAREFCAALRTYALCTRRTARTCRGDLAYHSAVHGIEDLMSQH	181<br>240
H. RGMA<br>D. RGMA	NCSKDGPTSQPRLRTLPPPGDSQERSDSPEICHYEKSFHKHAATPNYTHCGLFGDPHLRT<br>NCSKDGPTSQPRLRTLPPPGDSQERSDSPEICHYEKSFHKHAATPNYTHCGLFGDPHLRT	241<br>300
H. RGMA<br>D. RGMA	FTDRFQTCKVQGAWPLIDNNYLNVQVTNTPVLPGSAATATSKLTIIFKNFQECVDQKVYQ<br>FTDRFQTCKVQGAWPLIDNNYLNVQVTNTPVLPGSAATATSKLTIIFKNFQECVDQKVYQ	301<br>360
H. RGMA<br>D. RGMA	AEMDELPAAFADGSKNGGDKHGANSLKITEKVSGQHVEIQAKYIGTTIVVRQVGRYLTFA<br>AEMDELPAAFADGSKNGGDKHGANSLKITEKVSGQHVEIQAKYIGTTIVVRQVGRYLTFA	361<br>420
H. RGMA<br>D. RGMA	VRMPEEVVNAVEDRDSQGLYLCLRGCPLNQQIDFQAFRANAEGPGTRRQAAASPAPAAPD<br>VRMPEEVVNAVEDRDSQGLYLCLRGCPLNQQIDFQAFRANAEGPGTRRQAAASPAPAAPD	421<br>480
H. RGMA<br>D. RGMA	TFPYETAAAKCKEKLPVEDLYYQACVFDLLTTGDVNFTLAAYYALEDVKMLHSNKDRLHL<br>TFPYETAAAKCKEKLPVEDLYYQACVFDLLTTGDVNFTLAAYYALEDVKMLHSNKDRLHL	481<br>540
H. RGMA<br>D. RGMA	YERTQEPPGRAAAAALSPAPSPLLGSLLLLPMLPGLLEAMAA<br>YERTQEPPDRAAAAGLSPAPRPLLGSLLLLPMLPGLLEAMAA	541<br>600

Table S38. Comparative amino acid analysis of donkey and horse RGMB gene

H. RGMB<br>D. RGMB	MMRKKRKRGASPGPCRSHGPGPATAPAPPPSPEPTRSAWTGMGLRAAPSCAAAASAAAAG<br>MMRKKRKRGASPGPCRSHGPGPATAPAPPPSPEPTRSAWTGMGLRAAPSCAAAASAAAAG	1<br>60
H. RGMB<br>D. RGMB	AEQRRRPRLCPPPLALLLLLLLSLGLLHAGDCQQPAQCRIQKCTTDFVSLTSHLNSAIDG<br>AEQRRRPRLCPPPLALLLLLL-SLGLLHAGDCQQPAQCRIQKCTTDFVSLTSHLNSAIDG	61<br>120
H. RGMB<br>D. RGMB	FDSEFCKALRAYAGCTQRTSKACRGNLVYHSAVLGISDLMSQRNCSKDGPTSSTNPEVTH<br>FDSEFCKALRAYAGCTQRTSKACRGNLVYHSAVLGISDLMSQRNCSKDGPTSSTNPEVTH	121<br>180
H. RGMB<br>D. RGMB	DPCNYHSHTGAREHRGGDQTPPNYLFCGLFGDPHLRTFKDHFQTCKVEGAWPLIDNNYLS<br>DPCNYHSHTGAREHRGGDQTPPNYLFCGLFGDPHLRTFKDHFQTCKVEGAWPLIDNNYLS	181<br>240
H. RGMB<br>D. RGMB	VQVTNVPVVSGSSATATNKITIIFKAHRECTDQKVYQAVTDDLPAAFVDGSTSGGDGDTK<br>VQVTNVPVVSGSSATATNKITIIFKAHRECTDQKVYQAVTDDLPAAFVDGSTSGGDGDTK	241<br>300
H. RGMB<br>D. RGMB	SLRIVERESGRYVEMHARYIGTTVFVRQLGRYLTLAIRMPEDLAMSYEESQDLQLCVNGC<br>SLRIVERESGRYVEMHARYIGTTVFVRQLGRYLTLAIRMPEDLAMSYEESQDLQLCVNGC	301<br>360
H. RGMB<br>D. RGMB	PLGERIDDGQGQVSAILGHTLPRTSLTQAWPGYTLETANAQCHEKMPVKDIYFQSCVFDL<br>PLGERIDDGQGQVSAILGHTLPRTSLTQAWPGYTLETANAQCHEKMPVKDIYFQSCVFDL	361<br>420
H. RGMB<br>D. RGMB	LTTGDANFTAAAHSALEDVEALHPRKERWHIFPSSSRGTPRGGSRLSVSLGLTCLILIVF<br>LTTGDANFTAAAHSALEDVEALHPRKERWHIFPSSSRGTPRGGSRLSVSLGLTCLILIVF	421<br>480
H. RGMB<br>D. RGMB	L<br>L	481<br>540

Table S39. Comparative amino acid analysis of donkey and horse GREM1 gene

H.GREM1<br>D.GREM1	MSRTAYAVGALLLLLLGTLLPAAEGKKKGSQGAIPPPDKAQHNDSEQTQSPQQPGSRNRGR<br>MSRTAYAVGALLLLLLGTLLPAAEGKKKGSQGAIPPPDKAQHNDSEQTQSPQQPGSRNRGR	1<br>60
H.GREM1<br>D.GREM1	GQGPGTAMPGEEVLESSQEALHVTERKYLKRDWCKTQPLKQTIHEEGCNSRTIINRFCYG<br>GQGPGTAMPGEEVLESSQEALHVTERKYLKRDWCKTQPLKQTIHEEGCNSRTIINRFCYG	61<br>120
H.GREM1<br>D.GREM1	QCNSFYIPRHIRKEEGSFQSCSFCKPKKFTTMMVTLNCPELQPPTKKRRVTRVKQCRCIS<br>QCNSFYIPRHIRKEEGSFQSCSFCKPKKFTTMMVTLNCPELQPPTKKRRVTRVKQCRCIS	121<br>180
H.GREM1<br>D.GREM1	IDLD<br>IDLD	181<br>240

Table S40. Comparative amino acid analysis of donkey and horse GREM2 gene

H.GREM2	MLWKLSLSLFLVAVLVKVAEARKNRPAGAIPSPYKDGSSNTSERWQHQIKEVLASSQEAL	1
D.GREM2	MLWKLSLSLFLVAVLVKVAEARKNRPAGAIPSPYKDGSSNTSERWQHQIKEVLASSQEAL	60
H.GREM2	VVTERKYLKSDWCKTQPLRQTVSEEGCRSRTILNRFCYGQCNSFYIPRHVKKEEESFQSC	61
D.GREM2	VVTERKYLKSDWCKTQPLRQTVSEEGCRSRTILNRFCYGQCNSFYIPRHVKKEEESFQSC	120
H.GREM2	AFCKPQRVTSVLVELDCPGLDPPFRLKKIQKVKQCRCMSVNLSDSDKQ	121
D.GREM2	AFCKPQRVTSVLVELDCPGLDPPFRLKKIQKVKQCRCMSVNLSDSDKQ	180
